# Biodegradable Materials for Sustainable Health Monitoring Devices

**DOI:** 10.1021/acsabm.0c01139

**Published:** 2020-12-23

**Authors:** Ensieh S. Hosseini, Saoirse Dervin, Priyanka Ganguly, Ravinder Dahiya

**Affiliations:** Bendable Electronics and Sensing Technologies (BEST) Group, James Watt School of Engineering, University of Glasgow, G12 8QQ Glasgow, U.K.

**Keywords:** biodegradable materials, bioresorbable materials, naturally degrading sensors, implantable sensors, sustainable electronics, health monitoring

## Abstract

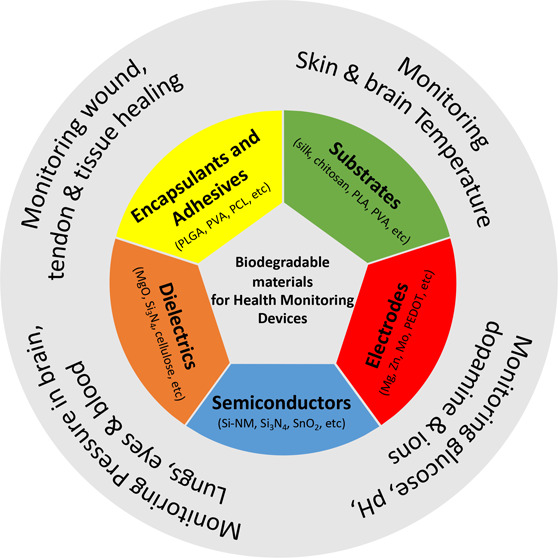

The recent advent of biodegradable materials has offered huge opportunity to transform healthcare technologies by enabling sensors that degrade naturally after use. The implantable electronic systems made from such materials eliminate the need for extraction or reoperation, minimize chronic inflammatory responses, and hence offer attractive propositions for future biomedical technology. The eco-friendly sensor systems developed from degradable materials could also help mitigate some of the major environmental issues by reducing the volume of electronic or medical waste produced and, in turn, the carbon footprint. With this background, herein we present a comprehensive overview of the structural and functional biodegradable materials that have been used for various biodegradable or bioresorbable electronic devices. The discussion focuses on the dissolution rates and degradation mechanisms of materials such as natural and synthetic polymers, organic or inorganic semiconductors, and hydrolyzable metals. The recent trend and examples of biodegradable or bioresorbable materials-based sensors for body monitoring, diagnostic, and medical therapeutic applications are also presented. Lastly, key technological challenges are discussed for clinical application of biodegradable sensors, particularly for implantable devices with wireless data and power transfer. Promising perspectives for the advancement of future generation of biodegradable sensor systems are also presented.

## 1 Introduction

Over the past decade, biodegradable sensors and electronic devices that naturally degrade or fully dissolve in their physiological environments have emerged as attractive alternatives for both invasive and noninvasive health monitoring.^1,2^ They provide a unique opportunity for temporary medical implants for continuous body condition monitoring and *in vivo* sensing. With flexible form-factors, such sensor systems integrated on wearables or clothing could offer a hygienic route for monitoring of various physiological parameters.^3−5^ The ability for real-time monitoring of parameters such as strain, pressure, temperature, pH, oxygen, and other specific biomarkers would expressively improve the information about tissue healing, early stage detection of postsurgical infection, and personalized treatments.^6−11^ Besides, they are ideal for single-shot measurements in point of care diagnostics as they are environmentally friendly and reduce the medical waste associated with disposable sensors and are usually low-cost.^12−16^

Most of the sensors that are used today in medical healthcare are often outside the body or noninvasive, and those implanted into the body have the disadvantage of removal surgery that exposes patients to the distress of retrieval and additional complications.^17,18^ Resorbable devices offering short-term performance are excellent temporary implants for diagnostic and therapeutic applications that need to operate only for a assigned duration and later elude without the need for surgical removal,^1^ thus promoting extensively to the patient’s comfort and eliminating the cost and risks of removal surgery. Additionally, using biocompatible and biodegradable materials for sensor fabrication minimizes the foreign body reactions to implants.

The main achievements in the development of biodegradable sensors are quite new and have started from about a decade ago with partially degradable sensors materials and recently with further advances in functional materials and fabrication methods, fully biodegradable systems that include power source, circuitry, and wireless technologies in flexible form-factors.^19^ Obtaining suitable materials for degradable sensors is challenging, considering that the materials they need to use should be nontoxic, biocompatible, biodegradable, and yet exhibit high-performance electrical/optical/mechanical properties. Flexibility and appropriate mechanical properties for conformal interfacing and minimal stresses on the tissue are other important aspects. The perfect route for such electronic devices requires production using green materials, those with renewable and abundant sources with intrinsic biocompatibility and biodegradability (such as protein derived or polysaccharide-based polymers), or synthetic materials with desirable properties found in common commodity products.

This review emphases on the major advancements in the area of biodegradable and implantable sensors used for monitoring of biomarkers and body signals for diagnostics and therapeutics. A comprehensive overview of the structural and functional biodegradable materials that have been used for the development of biodegradable systems, their properties, degradation mechanisms, and dissolution rates is presented in section 2. This includes active sensing materials, substrates, electrodes, interconnections, encapsulations, and adhesives layers. Section 3 presents some examples of recently developed physical and chemical biodegradable/bioresorbable sensors for *in vitro* body monitoring and those integrated into the body for diagnostic and medical therapeutic applications. In some medical applications, the biodegradable sensors are needed with wireless communication capability to decrease the chances of infection caused by wires breaching the skin. To achieve this, antennas/coils for data transmission and power supply have been explored. A few such examples have also been discussed in this section along with the potential opportunities they open for implanted sensors to enhance the understanding of biological systems. In section 4, the key current challenges associated with biodegradable sensors are discussed. This includes power requirements, size limitation, data transmission from the body, degradation kinetics of the device components, and performance stability. Lastly, the concluding section presents a summary of key conclusions, and the promising future perspectives for the advancement of a new generation of biodegradable devices, with emphasis on clinical application.

## 2 Structural and Functional Materials for Biodegradable Sensors

Motivated by the growing demand for degradable electronics, several research endeavors have targeted the expansion of biodegradable substitutes for conventional electronic components using safe, low-cost, large volume, and disposable materials to fabricate state-of-the-art biodegradable, bioresorbable, or transient devices. Subsequently, innovative approaches from materials science and engineering have facilitated the development of degradable fundamental device components including substrate materials, dielectric layers, active layers, conductive contacts and interconnects, and even circuitry using natural, synthetic, and conjugated biodegradable polymers, organic or inorganic semiconductors, and hydrolyzable metals.^20−25^

Polymeric materials offer high tunability in terms of their chemical structure, morphology, and dissolution time scale, the rate of which can be tailored by varying intrinsic polymer properties including molecular weight, crystal structure, chemical composition, hydrophilic or hydrophobic nature, and erosion mechanisms.^26−28^ In addition to flexibility and biocompatibility, this ability to tune the intrinsic properties of polymeric materials makes them promising candidates for compliant, customizable, and biodegradable device components. Among the wide range of naturally derived polymers, protein-based polymers, such as collagen, chitosan, fibrin, silk, and gelatin, as well as plant-based polysaccharides, including alginate, cellulose, dextran, and starch, have been increasingly employed for the fabrication of biodegradable devices. Many of these naturally derived biodegradable polymers also possess inherent bioactivity, due to similarities with biological macromolecules and can therefore elicit immunogenic response, if for example used as transient components for implantable healthcare technologies. Besides, weak mechanical properties, structural complexity, and high batch-to-batch variation often limit their use.^29−31^

Synthetic biodegradable polymers, on the other hand, offer greater control over the physiochemical properties of transient components and their resulting devices since they are produced under controlled and reproducible conditions.^30^ Since the simplest linear, aliphatic, and thermoplastic polyester, polyglycolide (PGA), was marketed as the first biodegradable suture in the 1960s, a range of synthetic biodegradable polymers numerous advancements have been made in the development of synthetic biodegradable polymers. Various biodegradable poly(α-esters) cross-linked elastomers, like poly(1,8-octanediol-*co*-citrictrate) and poly(glycerol sebacate) (PGS), polycarbonates, polyphosphazenes, polyurethanes (PU), polydioxanones, and polyhydroxyalkanoates (PHA) have since appeared.^29,32−36^ Among them, poly(lactic acid) or poly(lactide) (PLA/PLLA), poly(lactic-*co*-glycolic acid) or poly(lactide-*co*-glycolide) (PLGA), POC, PGS, poly(∈-caprolactone) or simply polycaprolactone (PCL), and more recently, polyhydroxybutyrate (PHB) and polyhydroxyvalerate (PHV) have attracted the greatest attention.^37−45^ The biocompatibility of these synthetic polymers has also been widely assessed.^46−51^ For instance, the biocompatibility of PLA and PLGA microspheres implanted in rats was verified using *in vivo* histological and immunologic analysis.^46^ The biocompatibility of PCL films has been demonstrated by examining the influence of their exposure on L929 mouse fibroblast viability.^47^ The biocompatibility of POC scaffolds was confirmed by their lack of influence on the morphology and phenotype of porcine chondrocytes.^48^ PGS membranes were also found to be biocompatible with both human cardiac mesenchymal stem cells and rat cardiac progenitor cells, supporting their adhesion and growth;^49^ PHB biocompatibility was evaluated through the inflammatory response of tissue after 4 and 12 weeks of subcutaneous implantation in rats.^50^ Finally, the *in vitro* and *in vivo* biocompatibility of silk has been extensively demonstrated through its widespread use as a passive substrate for biodevices, as well as its use in a number of biomedical applications, including drug delivery, wound healing, tissue engineering, and regenerative medicine.^51^

**Figure 1:**
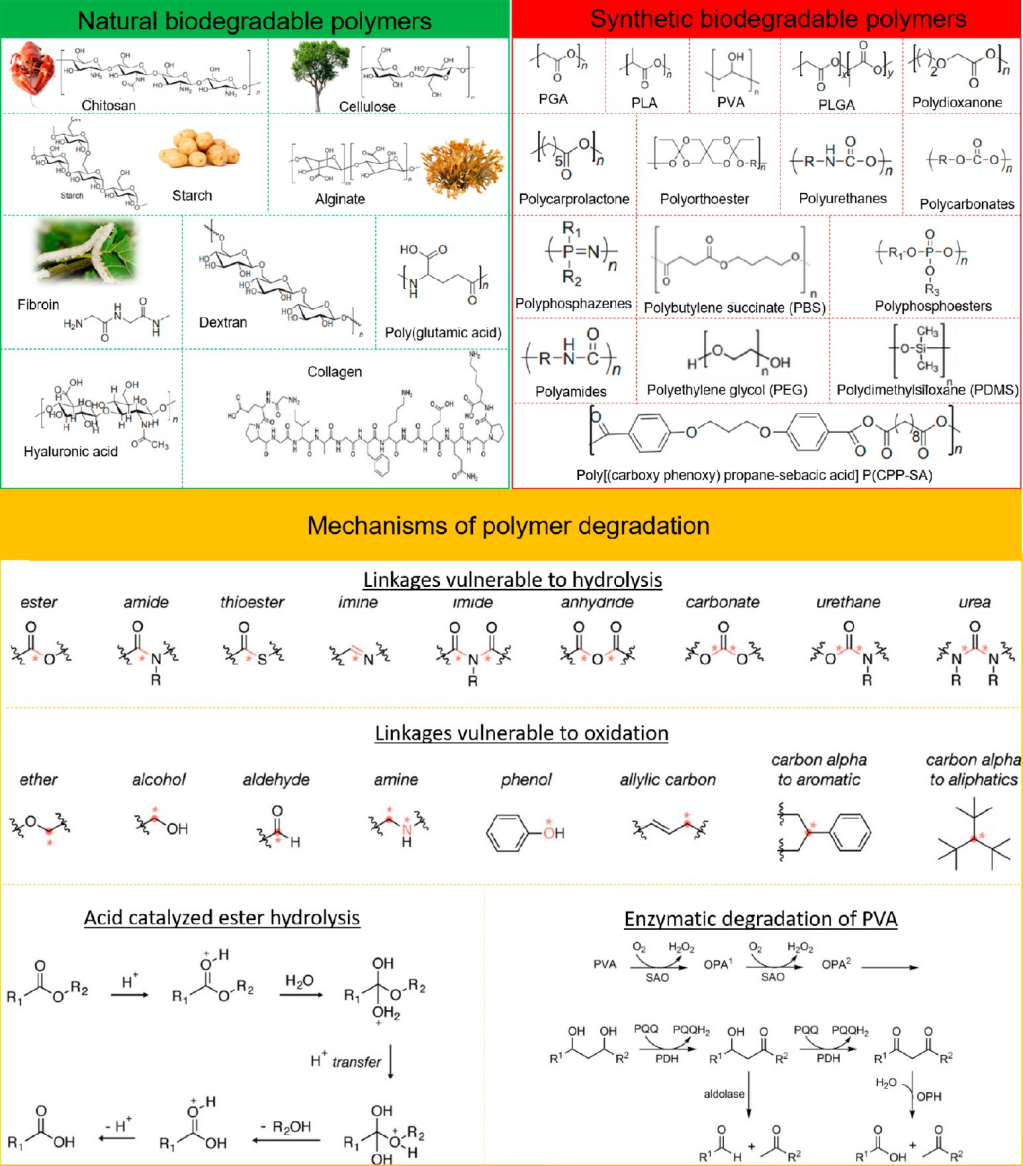
Chemical structures of natural and synthetic biodegradable polymeric materials. Chemical structures of moieties susceptible to hydrolysis and oxidation. Mechanism of acid-catalyzed ester hydrolysis. Mechanism of PVA enzymatic degradation. Reproduced with permission from refs (57 and 58). Copyright 2008 Woodhead Publishing and ref (59). Copyright 2014 WILEY-VCH Verlag GmbH and Co. KGaA, Weinheim.

The biodegradation of both naturally derived and synthetic polymers generally occurs through cleavage of unstable sites found along the polymer chain backbone, leading to a loss of polymeric materials.^52^ Many polymers derived from natural sources tend to undergo enzymatic breakdown by living organisms.^53^ However, in biological environments, biodegradation can also occur by hydrolysis, oxidation, or photooxidation.^53^ Synthetic polymers, on the other hand, are usually nonenzymatically degraded.^52^ The most widely used biodegradable synthetic polymers typically contain ester bonds that facilitate hydrolytic degradation in acidic or alkaline conditions.^54^ Amide, sulfonamide, anhydride, carbonate, ether, imide, imine, phosphonate, thioester, urea, and urethane bonds also serve as unstable sites susceptible to hydrolytic degradation (Figure 1).^23^ The rate of hydrolysis is largely based on the physiochemical characteristics of the polymeric material. For instance, as hydrophilicity or the frequency of hydrolyzable groups and the available surface area increase, so too does the rate of degradation.^55^ In contrast, increasing the cross-linking density and crystallinity, both of which limit the rate of water uptake, reduces the rate of hydrolysis.^55^ Environmental factors, for example, the temperature, pH, and the physiological composition of the surrounding environment also have a profound effect on the rate of hydrolysis. The degradation pattern of several known synthetic biodegradable polymers can therefore often be tuned to facilitate a desired transience time scale.^29,56^ Biologically relevant oxidative mechanisms, such as the release of reactive oxygen or nitrogen species by activated phagocytes during wound healing, can also facilitate the chemical or enzymatic cleavage of polymers (Figure 1).^57,58,55^ Take poly(vinyl alcohol) (PVA) for example. This synthetic highly polar, water-soluble polymer consists mainly of carbon atoms and repeating 1,3-diols units that can ultimately be broken down into acetic acid via microbial oxidation or enzymatic hydrolysis (Figure 1).^57,59^

In addition to biodegradable electronic polymer components, monocrystalline inorganic semiconductors, such as silicon (Si) nanomembranes (Si-NMs), are also considered vital materials for high-performance biodegradable devices due to their excellent operational characteristics and the well-established nature of Si semiconductor technologies.^60^ These chemically inert and biocompatible platforms also offer nanoscale thicknesses and, most importantly, undergo hydrolysis in biofluids.^12,21,22^ Recent research investigating the dissolution behavior of nanoscale elements of monocrystalline Si has greatly promoted their use in biodegradable electronics and highlighted the important roles of Si dopant type and concentration,^61−63^ temperature,^63^ pH,^62^ and the presence or concentration of proteins in the surrounding environment (Table 1).^64^ For example, increasing the temperature of the surrounding environment, as well as the concentration of chloride (Cl^–^) and phosphate (HPO_4_^2–^) anions, considerably increases the dissolution rate of Si-NMs through nucleophilic attack of Si surface bonds.^63^ Similarly, the presence of calcium (Ca) and magnesium (Mg) ions in phosphate-buffered saline (PBS) solutions can also increase the dissolution rates of Si.^64^ On the other hand, the absorption of proteins, like albumin, onto the Si surface reduces the rate of dissolution.^64^ Furthermore, when a certain degree of dopant concentration is exceeded (i.e., 1020 cm^–3^), the rate of Si-NM dissolution sharply decreases.^24^ The deposition condition of semiconductor thin films also significantly influences their physiochemical properties, which ultimately determines the dissolution rates of these materials.^65,66^ For instance, the dissolution of silicon oxides is 100-fold slower when using electron-beam (e-beam) deposition as opposed to plasma-enhanced chemical vapor deposition (PECVD). Likewise, nitrides deposited by low-pressure chemical vapor deposition (LPCVD) have slower dissolution rates than those deposited by PECVD. Related forms of Si, including polycrystalline Si (poly-Si), amorphous Si (a-Si), and germanium (Ge) and Si germanium (Ge) alloys (SiGe), have also demonstrated comparable dissolution rates to monocrystalline Si (Table 1).^67^ For example, poly-Si, a-Si, Ge, and SiGe in PBS solutions of pH 7.4 at 37 °C dissolve at rates of 2.8, 4.1, 3.1, and 0.1 nm day^–1^, respectively, in PBS solutions of pH 7.4 at 37 °C.^68^ a-Si dissolves faster than poly-Si due to an increased rate of water penetration, which stems from the lower activation energy and density of a-Si.^65,69,70^ Similarly, Ge has a lower bandgap and greater minority-carrier mobility than Si.^68^ Like monocrystalline Si, the dissolution of each of these materials is regulated by the temperature and pH of the surrounding environment as well as the presence of proteins and ions.^68^ For example, in comparison to room temperature, dissolution rates are accelerated at physiological temperatures (37 °C). In solutions of high pH (pH 10), patterned arrays of Si and Ge take only a few hours to dissolve at a pH of 10. Under similar conditions, however, SiGe dissolves at a much slower rate (∼2 nm day^–1^). This is because band-bending and a lowered potential barrier leads to large activation energy and the creation of a passivating oxide at the SiGe surface.^78^ Furthermore, the dissolution rates of poly-Si, a-Si, and nano-Si are 30–40-times higher in bovine serum than in PBS, while SiGe dissolved ∼185-times faster.

**Table 1:**
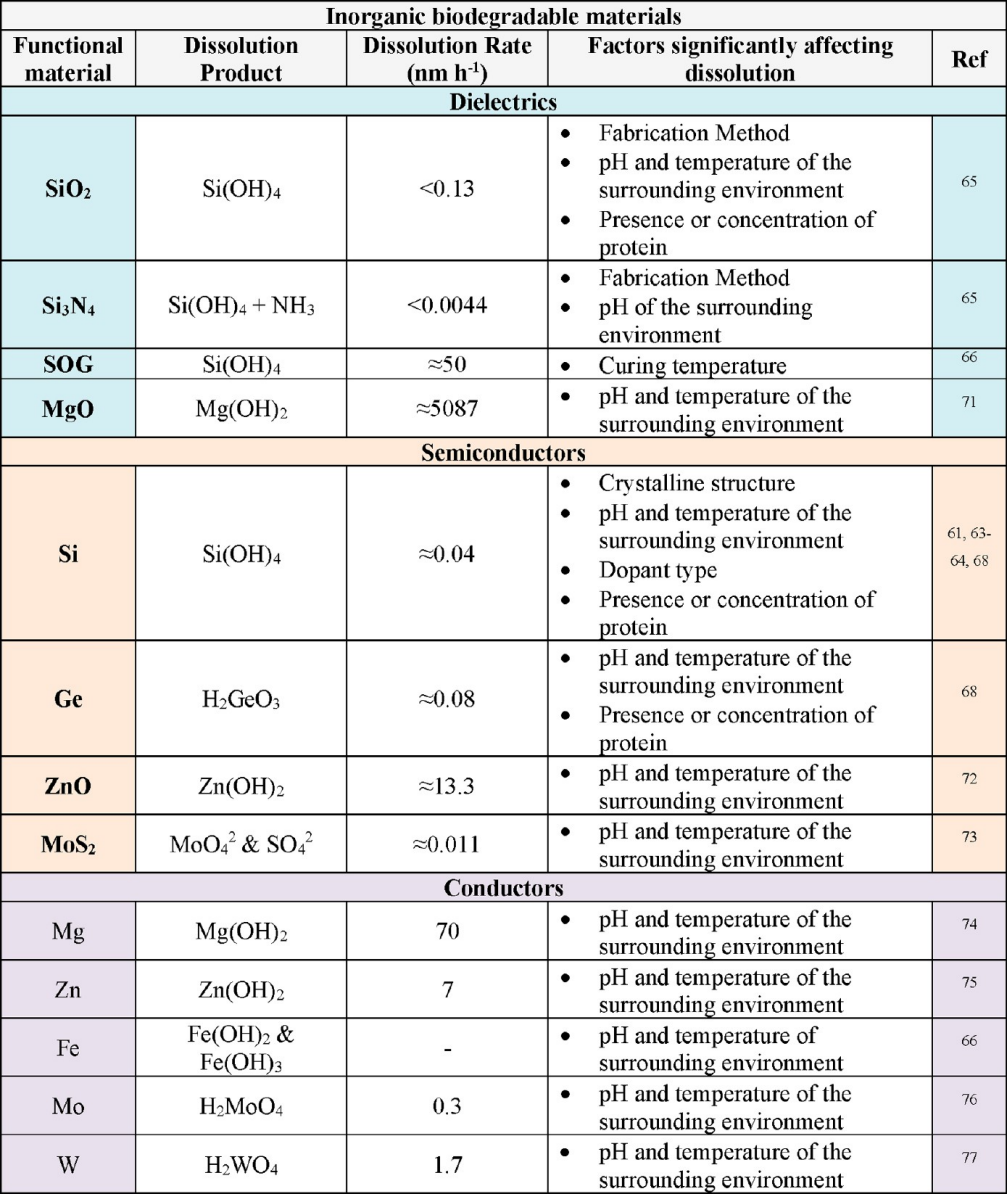
Dissolution Behavior of Inorganic Dielectric Materials, Inorganic Semiconductor Materials, and Hydrolyzable Metals in DI Water or Buffer Solution at Room Temperature^71,73,74,75,76,77^

Owing to their attractive electrical and mechanical performances, and their degradability in physiological environments essential metals and trace elements, including Mg, Molybdenum (Mo), Iron (Fe), Zinc (Zn), and their alloys, represent an exciting class of biomaterials for temporary medical device applications (Table 1).^70,79,80^ Because of their simplicity and fast dissolution rates (*r* = 7.2 nm day^–1^ and 115 nm day^–1^ in aqueous media and Hanks’s balanced saline solution, respectively), initial reports detailing biodegradable, bioresorbable, or transient devices components focused primarily on the use of Mg or Mg alloys as electrodes, interconnects, or structural device components.^12,23^ Mo, Zn, Fe, and Tungsten (W), however, share similar characteristics and transient mechanisms with Mg in both aqueous media and biofluids (Table 1).^70^ In addition, the biocompatibility of these biodegradable metals has also been widely assessed *in vivo* by examining inflammatory responses in both animal models and humans using histological and immunofluorescence analysis.^81−83^ However, in comparison to Zn, which has demonstrated dissolution rates of 1.7 nm day^–1^ in aqueous media and 7.2 nm day^–1^ in a biofluid, Mo and W are favored metals for temporary medical applications that may require direct contact between biological tissues and metals components, such as physiological electrical signal sensing, due to their slower, more tunable degradation (∼10^–2^ nm day^–1^). On the other hand, although both Mo and W have been deemed essential materials for the design of biodegradable healthcare devices, less comprehensive data relating to the biocompatibility of these metals and their degradation byproducts are available.^28,70^ Unique challenges are also faced when using biodegradable metals for ingestible or implantable electronics. For one, the physical properties of metal-based materials tend to change as they oxidize and dissolve.^84^ Besides, pockets of gas can form near implant sites due to excessive hydrogen evolution at the surface of metal contacts or interconnects. Since, however, ingestible electronics, like biodegradable antennae, will be used in the form of thin-film devices, excessive hydrogen evolution is not such a concerning risk. In addition, recent efforts to address potential challenges such as these have focused on the development of new composite alloys made up of Mg–Zn–Ca.^85^

Considering the large volume of suitable biodegradable materials that are available for the production of high-performance transient electronics, this section of the review will discuss recent developments in the selection of materials for the fundamental components of temporary biodegradable sensors for health monitoring.

### 2.1 Substrates

Electronic device substrates provide an electrically inert foundation for the deposition of multiple functional materials including dielectric layers, semiconductor materials, and conductive electrodes and interconnects. As a result, substrate area and thickness are larger than that of any other device layer. The substrate also constitutes most of the weight in an electronic device and therefore generates more “electronic waste” than the functional layers.^86^ In this respect, substrate materials ultimately determine device stability and degradation and are thus a critically important consideration.^23,24^ In consequence, the mechanical robustness, swelling rate, and dissolution rate are critical parameters for guiding the selection of suitable substrates for the design of high-performance biodegradable devices with controlled operational timeframes.^23,24^ To match device fabrication procedures, substrate materials must be compatible with high temperatures and harsh solvents..

#### 2.1.1 Naturally Derived Polymer Substrates

Recently, several protein-based biopolymers, including silk, cellulose, and chitosan, etc., have attracted extensive attention as substrate materials for biodegradable devices because of their readily abundant availability, outstanding biocompatibility, flexibility, and environmental sustainability.^24,87−90^

**Figure 2:**
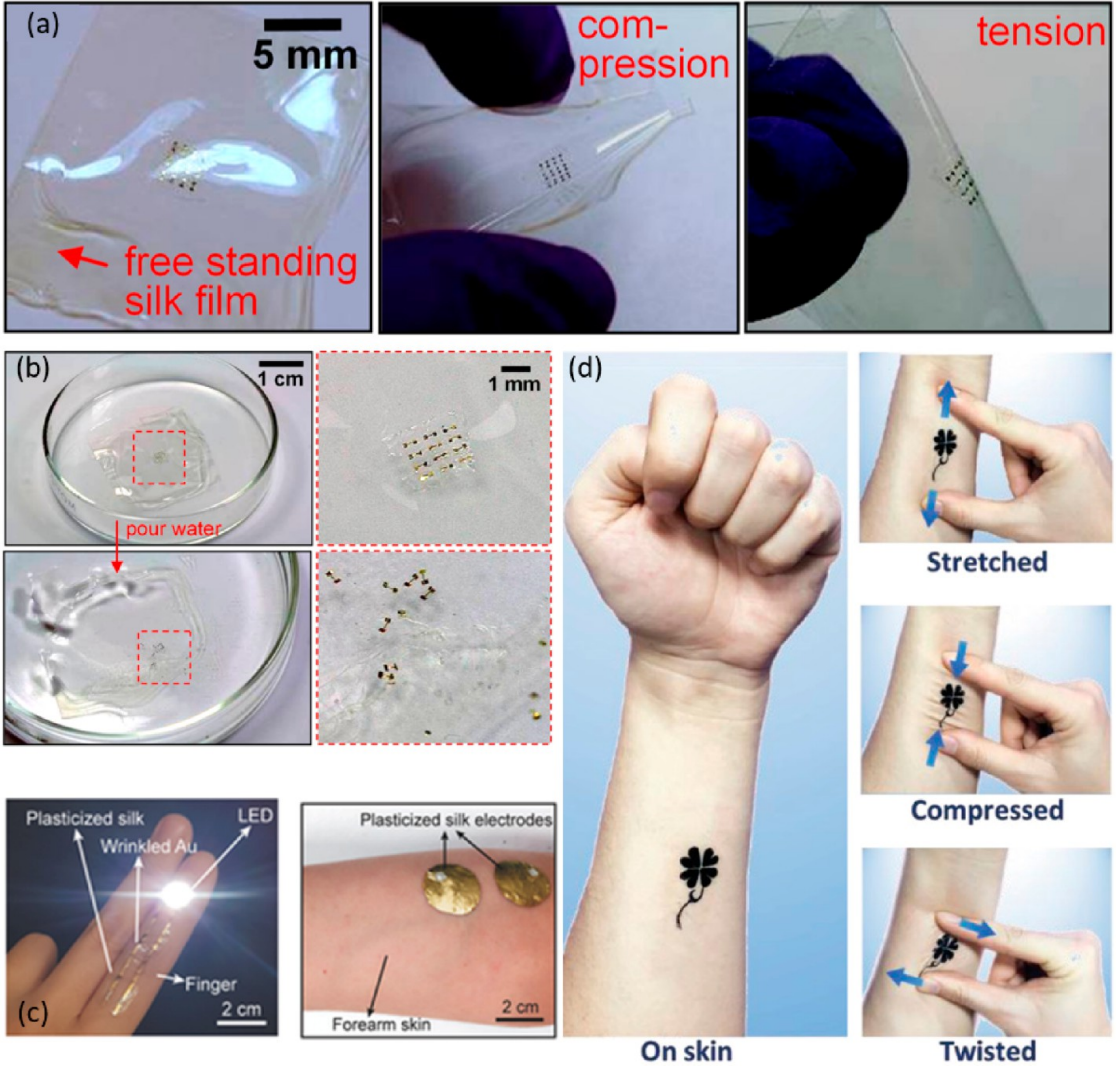
(a) Ultrathin devices on a flexible silk substrate, in flat (left) and bent (center and right) configurations, and (b) images of the water dissolution of silicon electronics on silk, at various time stages. Reproduced with permission from ref (19). Copyright 2009 American Institute of Physics (AIP). (c) Photographs of plasticized silk electrodes conformably attached to a finger and laminated on a human forearm. Reproduced with permission from ref (97). Copyright 2018 WILEY-VCH Verlag GmbH and Co. KGaA, Weinheim. (d) Photographs showing a silk-graphene E-tattoo attached to the forearm and the tattoo on skin in a stretched (upper), compressed (middle), and twisted (lower) state. Reproduced with permission from ref (98). Copyright 2019 WILEY-VCH Verlag GmbH and Co. KGaA, Weinheim.

Over the past two decades, the mechanical robustness, bioactivity, excellent biocompatibility, and immuno-compatibility of silk and regenerated silk fibroin materials have led to increased recognition of these biomaterials as a distinct class of biodegradable and biocompatible polymers for transient health monitoring technologies.^30^ Besides, the well-characterized degradation rate of these US Food and Drug Administration (FDA) approved biomaterials can be regulated via controlled β-Sheet crystallization. On the other hand, as crystallinity increases, silk becomes less flexible, more brittle, and difficult to handle. In addition, the rapid degradation rate of silk renders this fibrous protein incompatible with aqueous processing steps, which typically limits the potential for direct device fabrication.^91^ However, if the complete electronic system of a transient device is first fabricated on temporary substrate and then transferred to the substrate chosen for device operation, almost any biodegradable material can be used as a structural support for soft transient electronic devices.^86^ Using such approaches, silk has been demonstrated as a suitable support for a new generation of mechanically flexible and degradable Si-based implantable electronics with bespoke *in vivo* lifetimes (Figure 2a,b).^19^ The dissolution process, which can be tailored from periods of months to even years, relies on complex mechanisms that are ordinarily arbitrated by a foreign body response.^92^ Typically, silk is first broken down by proteolytic enzymes, for example, chymotrypsin, actinase, and carboxylase, via adsorption at surface-binding domains, followed by hydrolysis of the ester bond.^93^ The last degradation products include noninflammatory amino acids, which are simply absorbed *in vivo* and often usable in cell metabolic functions. Similar advances have also led to ultrathin biointerfaced electronic systems that provde intimate contact and minimal invasiveness for integration with the soft curved profiles of biological tissues.^94^ Silk has also been used to support the biotransfer of peptide modified, transfer printed graphene nanosensors onto biomaterials, with tooth enamel for the bioselective detection of bacteria. Biotransfer of the graphene nanosensors was achieved via dissolution of the water-soluble silk substrate.^95^ Other approaches have described the fabrication of biodegradable, flexible, and optically transparent, all organic micropatterned bioelectronic devices via aqueous photolithographic processing of conducting polymers (CPs) on silk substrates.^96^ Skin-conformable stretchable electrodes have also been developed for wearable and implantable applications using silk plasticization and thin-film metallization (Figure 2c).^97^ These highly stretchable (>100%) plasticized silk electrodes demonstrated excellent electrical on-skin electrophysiological signals recording performance, comparable to commercial gel electrodes. More recently silk was combined with graphene to serve not only as a substrate but also as an electrically conductive path for the realization of self-healing, skin mountable electronic tattoos that are sensitive to changes, in strain, temperature, and humidity (Figure 2d).^98^

**Figure 3:**
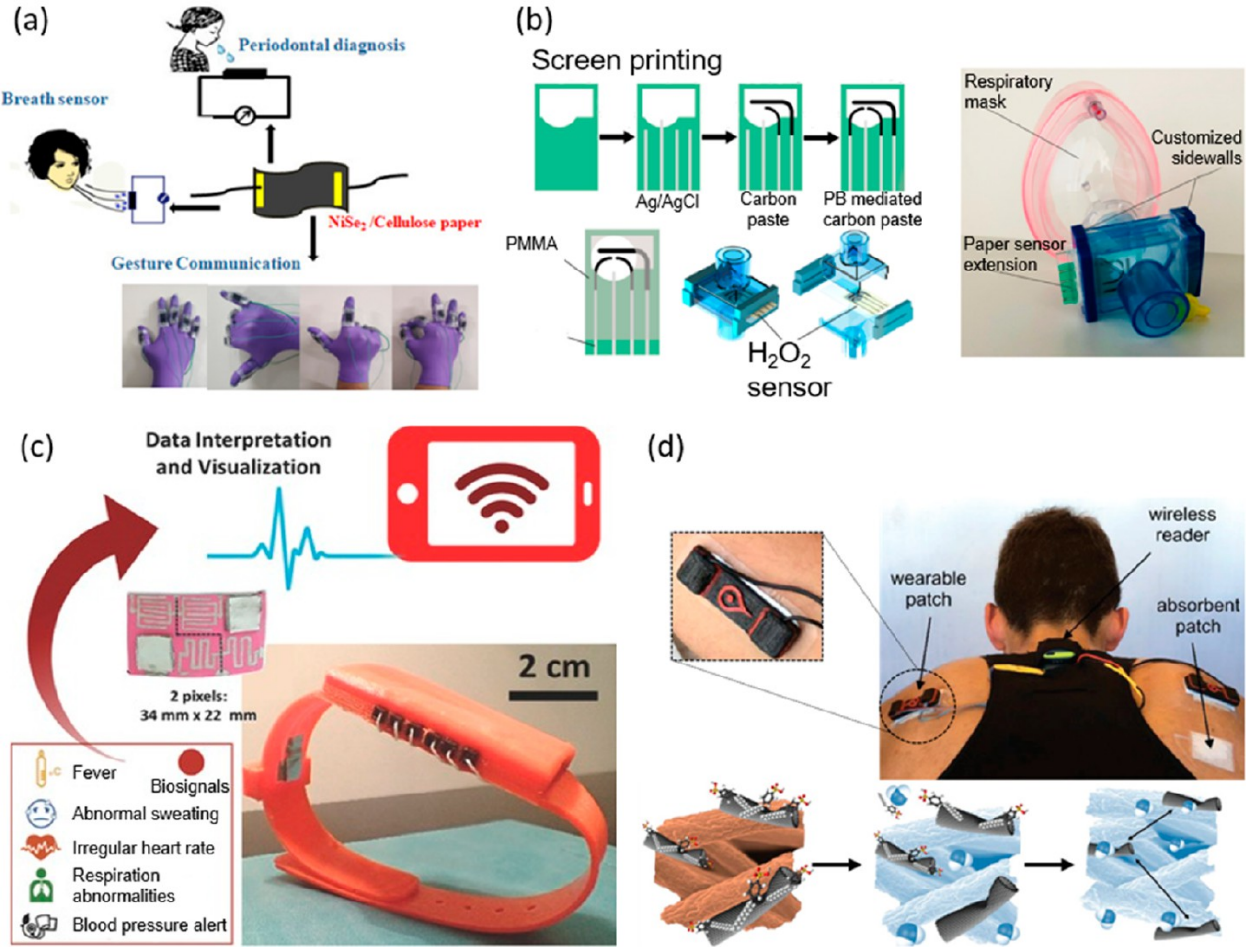
(a) Schematic diagram demonstrating the fabrication of a noninvasive periodontal diagnostic sensor, a breath analyzer, and a gesture sensor using NiSe_2_ modified cellulose paper. Reproduced with permission from ref (103). Copyright 2019 American Chemical Society. (b) Schematic diagram demonstrating the fabrication of a real-time paper-based H_2_O_2_ sensing chip and image of a respiratory mask that includes customized sidewalls and an extension of a commercial filter with the paper-based sensor. Reproduced with permission from ref (104). Copyright 2019 American Chemical Society. (c) Integration of a 3D stacked paper-based autonomous healthcare monitoring system. Reproduced with permission from ref (105). Copyright 2017 WILEY-VCH Verlag GmbH and Co. KGaA, Weinheim. (d) Paper-based sweat sensor for human perspiration monitoring. Reproduced with permission from ref (106). Copyright 2019 WILEY-VCH Verlag GmbH and Co. KGaA, Weinheim.

Aside from silk, cellulose, the utmost available natural polysaccharide on Earth, is also a promising substrate for biodegradable sensors for healthcare monitoring due to the complex carbohydrate’s attractive degradation behavior in physiological environments as well as its flexibility, transparency, high-temperature stability, and excellent biocompatibility.^99−101^ Conversely, the potential fabrication of ultrathin cellulose-based devices, a desirable characteristic for enhanced flexibility and faster degradation, is limited by the substrates tendency to exceed multiple micrometers in thickness. To overcome such issues, trimethylsilyl-functionalized cellulose was spin-coated on a thin dextran sacrificial layer to produce a cellulose substrate with good chemical and thermal stability and thickness as low as 800 nm.^91^ In a similar regard, papers made of cellulose nanofibers (CNF) have also been recognized as affordable, green biobased platforms for the fabrication of low-cost devices and biosensors for healthcare diagnostics. Paper is also a recyclable and eco-friendly household material that has been in use in our daily lives for centuries, and thus, its manufacturability, simplicity, and authenticity are beyond doubt. In addition, this simple substrate is versatile, flexible, and porous and can thus facilitate the accurate and rapid detection of targeted physiological analytes. By this means, paper-based devices can provide inexpensive and transportable diagnostic technologies that can be immensely useful in resource-constrained settings, where special instrumentation and medical professionals are not always readily accessible.^102^ For instance, due to its high processing temperature (275 °C), paper was chosen as a suitable substrate to support the large area growth of NiSe_2_ for the fabrication of a pH sensor for noninvasive monitor oral health monitoring, a breath sensor to monitor breath-related diseases, and a physical strain sensor for gesture recognition, to assist deaf, dumb, and aurally challenged (Figure 3a).^103^ Disposable, lightweight, and low-cost paper-based, “calibration-free” electrochemical wearable sensors have also been established for real-time, continuous, and on-site testing of exhaled hydrogen peroxide (H_2_O_2_) in artificial breath (Figure 3b).^104^ These versatile electrochemical paper-based sensors can also be easily incorporated within a commercial respiratory mask. The flexible and hygroscopic porous paper acted as both a support for screen-printed electrodes and as a “solid electrolyte”, eradicating the requirement for additional membranes. In addition, both the sensing surface and collection volume of the device could be significantly improved by shaping or patterning the paper-based substrate. A “paper watch” has also been developed for simultaneous and real-time detection of body vital conditions including blood pressure, heart rate, body temperature, and skin hydration, using recyclable and nonfunctionalized using Post-It paper as a structural support (Figure 3c).^105^ By using Post-It paper as a substrate, the ultralow-cost wearable health monitoring system presents a simple approach for integration, a small environmental footprint, and improved contact intimacy with the skin. In addition, a wearable paper-based chemiresistor for assessing both sweat rate and sweat loss in the human body has been fabricated by integrating a nanocomposite of single-walled carbon nanotubes (SWCNTs) and sodium dodecylbenzenesulfonate surfactant within the cellulose fibers of commercial filter paper (Figure 3d).^106^ The resulting wearable device provides simple and cost-effective, real-time perspiration measurements that could be used for several on-body biofluid analysis applications.

**Figure 4:**
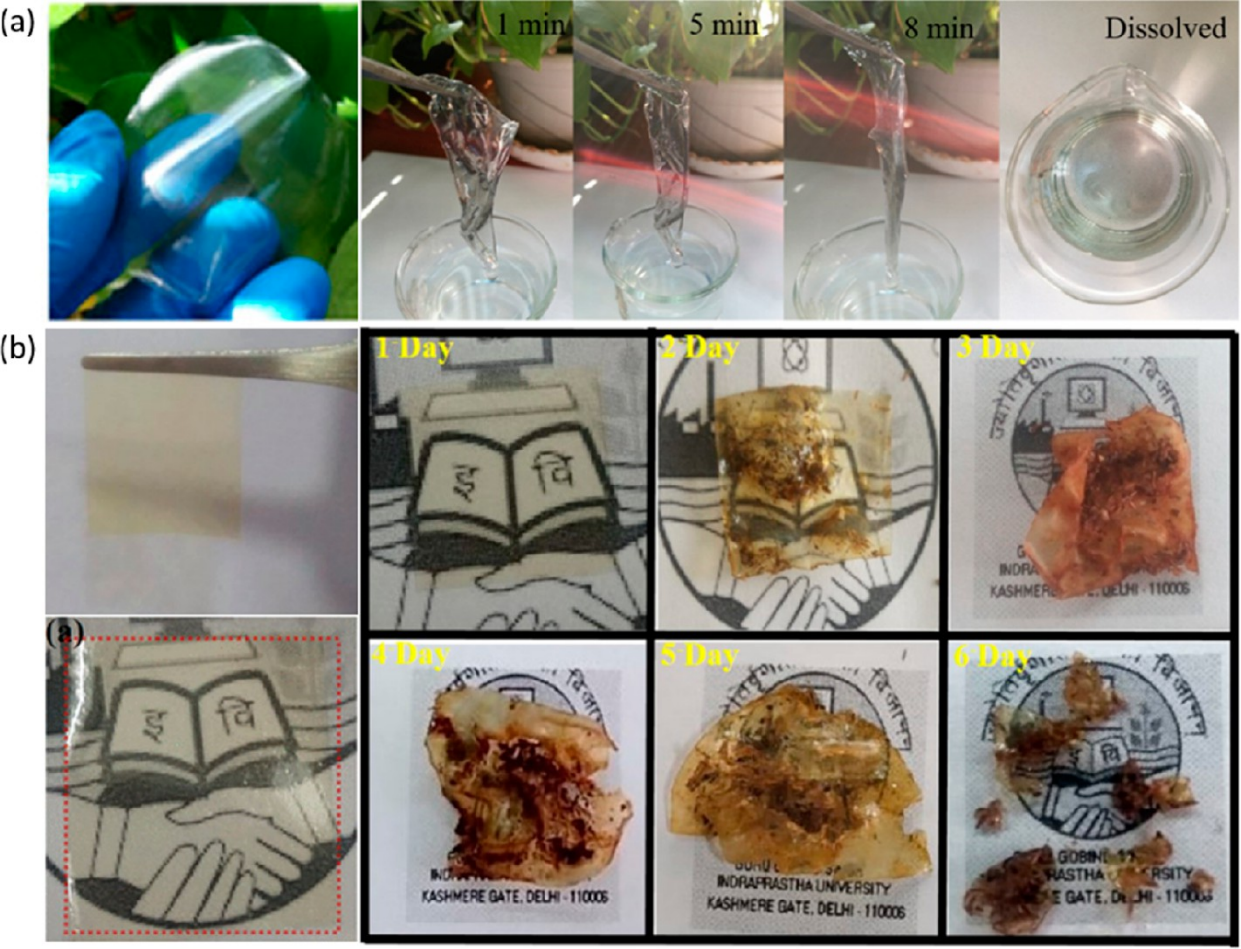
(a) Photographs of a flexible and transparent starch–chitosan substrate (SC) and dissolution of an SC based transparent electrode. Reproduced with permission from ref (113). Copyright 2018 American Chemical Society. (b) Photographs of a chitosan–PVP substrate and its biodegradability in soil. Reproduced with permission from ref (115). Copyright 2019 American Chemical Society.

After cellulose, chitin, the structural polymer commonly found in the shells of crabs, shrimp, lobster and squid, as well as some mushrooms, green algae, molds, and yeast, is the second most abundant biopolymer.^107,108^ Because of its highly ordered crystalline structure and corresponding lack of solubility, however, the use of chitin, in many cases is limited.^109^ On the other hand, the N-deacetylated derivative of chitin, chitosan, a linear cationic polysaccharide that consists of glucosamine and *N*-acetyl-glucosamine, is soluble in aqueous solutions of both organic and inorganic acids. Chitosan is also biodegradable and biocompatible and has functions of antibacterial, anti-inflammatory, and hemostatic activity.^110,111^ These unique features thus make chitosan an ideal candidate for a multitude of diverse biomedical applications, for example, as FDA approved wound dressings to promote healing or for the local management of bleeding wounds, bioscaffolds for epithelial and soft tissue engineering, and for drug delivery systems.^9,112^ Despite this, chitosan’s inadequate mechanical properties often limit this natural substrate’s use in a wide variety of applications. In consequence, chitosan is often mixed with other polymers to enhance its properties and further diversify its applications. For example, by blending chitosan, extracted from crab shell with another widely abundant and naturally sourced polymer, potato starch, wearable green electronics based on cheap, edible, biodegradable, transparent and water-soluble substrates have been developed (Figure 4a).^113^ The edible starch–chitosan substrate-based transparent electrodes can be biodegraded in lysozyme solution quickly at room temperature, deprived of creating any toxic remains. Our group has also recently developed a free-standing, biodegradable, piezoelectric film for the development of fully degradable pressure sensing devices by blending chitosan with bioorganic glycine.^7,114^ Chitosan has also been blended with synthetic polymers, like poly(vinylpyrrolidone) (PVP), to fabricate biodegradable, low-cost, and flexible substrates (Figure 4b).^115^ The chitosan–PVP flexible substrate demonstrated high optical transmittance, high-temperature stability, smooth surface, and good mechanical stability. The natural–synthetic polymer blend also revealed a high degree of biodegradability, degrading by almost ∼90% of its original state after just 6 days in farmland soil at room temperature.

A variety of additional natural compounds, including bio-organics, such as sodium alginate and even foodstuffs, like charcoal, rice paper, potato starch, gelatin seaweed, and cheese, have also been used as natural substrates for biodegradable devices.^22,116^

#### 2.1.2 Synthetic Polymer Substrate

Although natural substrate materials present an exciting platform for transient technologies, their intrinsic properties often fail to meet the high demand drawn by biodegradable electronic devices.^23^ For instance, the mechanical properties and rate of degredation of naturally derived substrates are often hard to tune. Natural polymers also have the capacity to elicit immune responses.^117−119^ In addition, the increasing demand for improved integration of biodegradable devices with dynamic surfaces, such as wearable or implantable transient electronics designed to adhere to the skin, heart, or brain, further stresses substrate requirements to consider stretchable and elastic features.^120,121^ In contrast, many naturally derived biopolymers tend to demonstrate a brittle nature, particularly as crystallinity and thickness increase.^23^ Instead, synthetic polymers with foreseeable and reproducible mechanical and disintegration performances can be produced using controlled conditions.^119,122−124^ Because of these advantages and the increasing interest in green electronics, several synthetic polymers with unique features, including PLA, PLGA, PU, PVA, PCL, poly(caprolactone)–poly(glycerol sebacate) (PGS–PCL), poly(ethylene glycol) (PEG), polydimethylsiloxane (PDMS), polybutylene succinate (PBS), and sodium carboxymethylcellulose (Na–CMC) are gaining prominence as substrates for soft, elastic transient electronics.^125−127^

**Figure 5:**
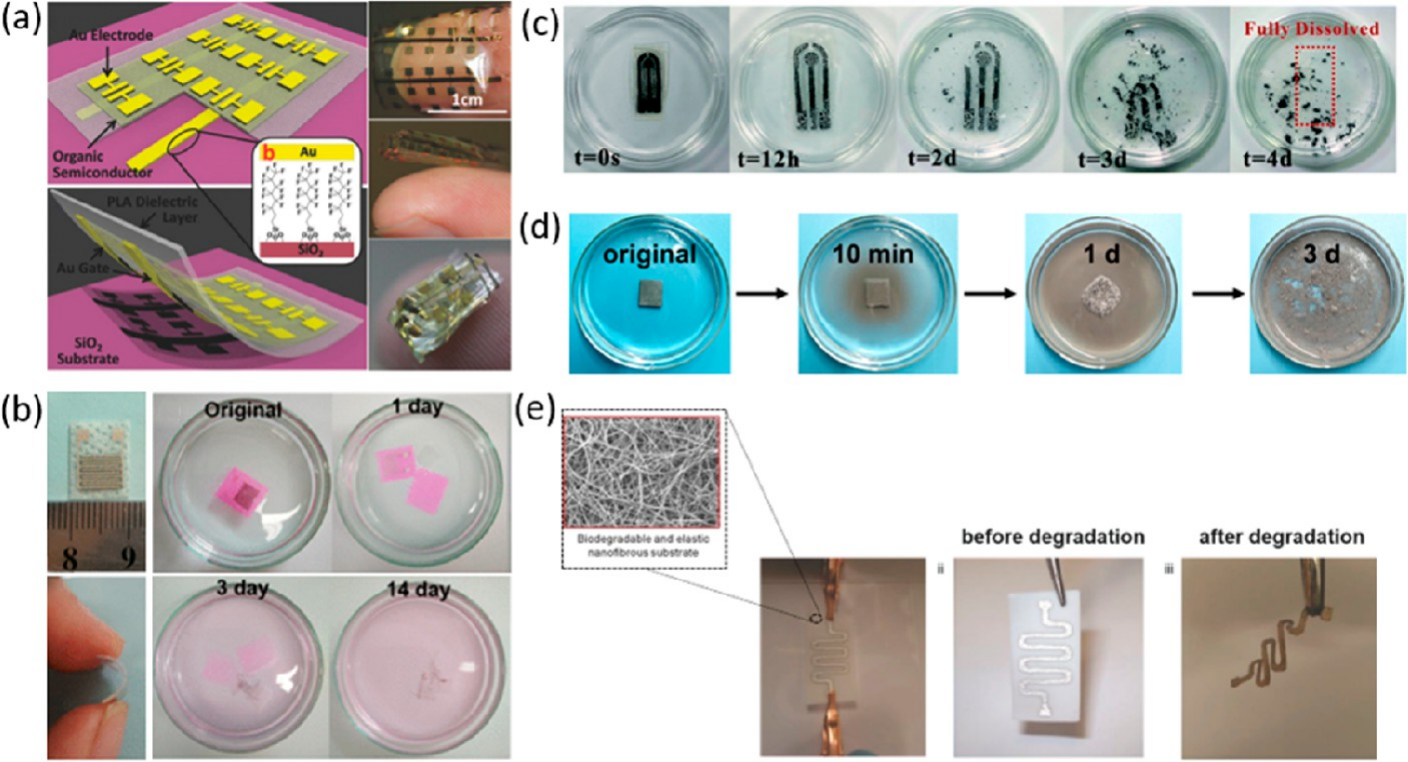
(a) Schematic diagram demonstrating the fabrication of an OFET device using a three-arm stereocomplex PLA substrate and images of the fully constructed transparent and flexible OFETs. Reproduced with permission from ref (132). Copyright 2015 WILEY-VCH Verlag GmbH and Co. KGaA, Weinheim. (b) Photographs of a flexible wearable transient MXene/tissue paper sensor sandwiched between a PLA thin sheet and an interdigitated electrode-coated PLA thin sheet, and its dissolution over 2 weeks in a 0.5 M NaOH solution. Reproduced with permission from ref (133). Copyright 2019 American Chemical Society. (c) Sequential dissolution images of a fully transient PVA-based electrochemical strip in DI water. Reproduced with permission from ref (135). Copyright 2020 The Royal Society of Chemistry. (d) Photographs of a PVA–liquid metal hydrogel for wearable transient epidermal sensors placed in a HCl solution. Reproduced with permission from ref (136). Copyright 2019 American Chemical Society. (e) Images showing the degradation of a patterned electrospun nanofibrous PGS–PCL substrate inside a solution of 0.5 M NaOH. Reproduced with permission from ref (127). Copyright 2014 WILEY-VCH Verlag GmbH and Co. KGaA, Weinheim.

Of the many synthetic polymer substrates available for the fabrication of biodegradable healthcare monitoring devices, PLA is a commercially attractive due to its similaritites with traditional hydrocarbon polymers such as polyethylene terephthalate (PET), polystyrene (PS), and polycarbonate (PC). In addition, PLA can be produced from lactic acid by direct polycondensation reaction, or ring-opening polymerization of the lactide monomer. This means that, while PLA is a synthetic polymer, this biodegradable thermoplastic polyester can be derivative of several renewable resources including corn starch, tapioca roots, or sugar cane.^128^ Furthermore, the FDA has also permitted the use of PLA in specified clinical applications.^129,130^ In consequence, PLA, in addition to PLLA and PLGA, has been used for the manufacture of biodegradable disposable products, as bioresorbable biomaterial substrates, scaffolds, and medical implants, and for drug-delivery systems.^131^ For example, a three-arm stereocomplex PLA (tascPLA) has been employed as both a dielectric and substrate material for the fabrication of a skin-like temperature sensor array created on a highly flexible and thermally stable (up to 200 °C) organic transistors (Figure 5a).^132^ The biomaterial-based organic field-effect transistors (OFETs) present many advantages, including transparency, degradability, reliable skin-like thermal sensitivity, and good biocompatibility, hence displaying wide ranging applicability for implantable medical devices and artificial skin as well as environmentally friendly electronics. Flexible wearable transient pressure sensors to act as an electronic skin (e-Skin) for mapping tactile stimuli and to forecast the potential health condition of patients have also been developed by inserting porous MXene-impregnated tissue paper between two PLA thin sheets, one coated with an interdigitated electrode.^133^ Because of the advantageous degradability of both PLA and tissue paper, the sensor degraded after just 14 days in 0.5 M NaOH (Figure 5b).

PVA is another popular substrate choice for biodegradable and biocompatible electronic devices. For example, PVA has been used as a temporary substrate to support sensors, transistors, light-emitting diodes, photodetectors, radiofrequency inductors, capacitors, oscillators, rectifying diodes, wireless coils, and solar cells on thin PDMS foil for the measurement of electrical activity produced by the heart, brain, and skeletal muscles.^134^ Like other polymer materials, both natural and synthetic, PVA is often mixed with blend polymers to achieve improved material properties and targeted or tunable device characteristics. For instance, research has previously shown that the addition of gelatin to a PVA–polymer matrix achieved the necessary film strength and transience required for long-term point of care glucose levels using electrochemical test strips.^135^ Although bare PVA films also maintained their shape during electrochemical analysis, their dissolution was timely. In comparison, PVA–gelatin substrates fully degraded after 7 days in deionized water at room temperature (Figure 5c). PVA-based hydrogels have also been used as a platform for the development of transient wearable sensors for healthcare diagnosis and to monitor human activity. For instance, borate-modified PVA hydrogels stabilized with liquid metal particles (LMPs) of eutectic gallium and indium were fabricated as epidermal sensors.^136^ The PVA–LMP-based sensors demonstrated attractive dissolvable features for on-demand transient electronics, largely dissolving after just 3 days in pH 5 HCl solution at room temperature (Figure 5d). The excellent degradation properties were ascribed to the separation of the dynamic diol–borate complex and destruction of the 3D hydrogel network in acidic environments or disrupted liquid inner cores due to acid attacking the oxidized shells of LMPs.

PGS, a biodegradable elastomer, is yet another excellent candidate for temporary health monitoring devices that can withstand up to ∼30% strain with linear elastic mechanical responses.^137^ Electrospun elastic PGS–PCL substrates have been used to engineer stretchable and biodegradable electronics to serve as elastic and biocompatible heaters, temperature sensors, and strain gauges for bioresorbable electronics and smart wound dressings. Electrospun PGS–PCL substrates are generally constructed of a thin fibrous mesh and present wicking properties similar to paper. This enables their compatibility with fabrication processes normally applied in paper electronics.^127^ These processes range from using screen printing or inkjet printing of metallic inks to patterning or machining using a laser to deposit electrically conductive traces.^138^ These substrates also offer elasticity, suturability, and gradual degradability. After 10 days in 0.01 M NaOH and PBS solutions at 37 °C, the variation in the electrical resistance of serpentine silver lines patterned on the nanofibrous substrates varied by less than 10%.^127^ After 30 days, however, the silver patterned PGS–PCL substrate had completely degraded, highlighting the material’s potential as a substrate for bioresorbable electronics and smart wound dressings (Figure 5e).

Although biodegradable synthetic polymer substrates offer superior mechanical properties over their natural counterparts, they too are often incompatible with direct device fabrication techniques.^139^ In this regard, transfer printing techniques can be used to avoid the constraints associated with the inherent features of synthetic biodegradable polymers. For instance, transient complementary metal-oxide–semiconductor (CMOS) arrays, with excellent operational characteristics, were fabricated on several synthetic biodegradable substrates including PLGA, PCL, and rice paper. Ultrathin Si microdevices were first deposited, patterned, and etched, and then transferred to the desired biodegradable substrate using transfer printing.^86^ To demonstrate the potential of this manufacturing strategy, a fully formed transient hydration sensor that might be used to monitor the healing processes of cutaneous wounds was transfer printed from a temporary substrate where it was formed onto a PLGA substrate. Critically, the performance of the transfer printed device was comparable to that of devices before transfer. Under physiological conditions, in PBS at 37 °C, the dissolvable inorganic components the PLGA-based device required only a matter of days or weeks to degrade, while the PLGA substrate took some months. This difference in degradation times could, therefore, be used to develop robust yet transient sensing systems that rely on the degradation of inorganic elements as the sensing mechanism. Similar transfer printing techniques have also been reported for the development of biodegradable Si-based devices on PVA substrates.^139−141^

#### 2.1.3 Metal Substrates

As alternatives to biodegradable polymeric substrates, metal foils, including Mo, Fe, W, and Zn, have also served as substrates for biodegradable devices. For instance, n-channel metal–oxide–semiconductor field-effect transistors (MOSFETs) have been fabricated on a Mo foil (≈ 5 μm) to serve as transient active and passive electronic components including diodes, transistors, capacitors, and inductors. The transient n-MOSFETs completely dissolved after 25 days in PBS (pH 7.4) at 90 °C. Because of excellent electrical and thermal properties, relative solvent resistant, and favorable water and oxygen isolation performances, metal foils such as these provide improved compatibility with device manufacture techniques.^66^ They also avoid the issue of polymer swelling in aqueous solutions. On the other hand, their rigid properties often limit use in a varied range of applications.

### 2.2 Dielectric Materials

Dielectric materials are electrical insulators that generate a large polarization in the presence of an electric field. Because of the alignment of dipole moments in an external electric field, positive charges present in the dielectric material move in the direction of the applied field and negative charges move opposite to this, resulting in an internal electric field that decreases the overall field that is contained by the dielectric. Dielectric materials are, therefore, integral components of both active and passive devices such as field-effect transistors (FETs) and capacitive sensing devices, both of which contribute significantly to the realization of medical diagnostics and structural health monitoring devices.^23,125^

#### 2.2.1 Inorganic Dielectrics

**Figure 6:**
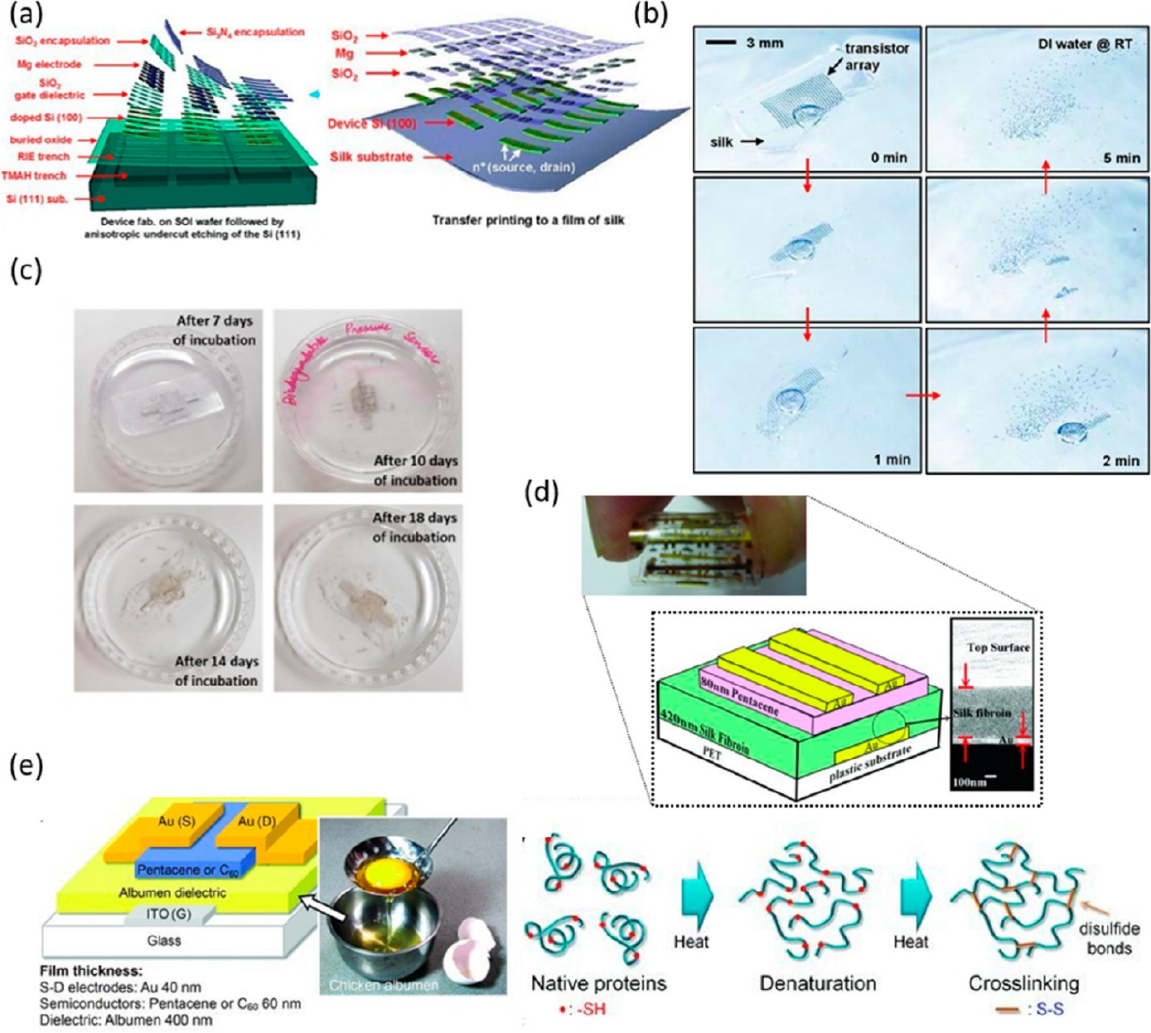
(a) Schematic diagram representing the wafer-scale fabrication of fully formed transient n-channel MOSFETs based on SiO_2_ gate and interlayer dielectrics, and subsequent transfer of the device to silk films. (b) Optical images of the dissolution and disintegration of an array of MOSFETs on silk. Reproduced with permission from ref (142). Copyright 2013 WILEY-VCH Verlag GmbH and Co. KGaA, Weinheim. (c) Images of the degradation of a highly sensitive biodegradable pressure sensor based on nanofibrous PLGA–PCL dielectric in PBS. Reproduced with permission from ref (147). Copyright 2019 Elsevier. (d) Rollable pentacene OTFT with silk fibroin as the gate dielectric. Reproduced with permission from ref (165). Copyright 2011 WILEY-VCH Verlag GmbH and Co. KGaA, Weinheim. (e) Schematic diagram representing the structure of an OFET fabricated using egg white as a dielectric and a schematic diagram demonstrating the denaturation and cross-linking of albumen protein under thermal treatment. Reproduced with permission from ref (178). Copyright 2011 WILEY-VCH Verlag GmbH and Co. KGaA, Weinheim.

Inorganic dielectric materials, such as magnesium oxide (MgO), silicon dioxide (SiO_2_), silicon nitride (Si_3_N_4_), and spin-on-glass (SOG) represent attractive choices for gate and interlayer dielectrics, passivation coatings, and the encapsulation layers of biodegradable devices to avert short circuit and shield other functional materials.^22,66^ SiO_2_ and Si_3_N_4_ are two of the most used dielectric materials. For example, n-channel monocrystalline silicon MOSFETs have been developed for biodegradable electronic implants using silk substrates (≈ 25 μm), Si semiconductors (≈ 100 nm), Mg source, drain, and gate electrodes (≈ 200 nm), and SiO_2_ gate (≈ 100 nm) and interlayer (≈ 100 nm) dielectrics (Figure 6a).^142^ The constructed devices demonstrated on/off ratios of >10^5^ and mobilities of 650 cm^2^/V s. Furthermore, the free-standing MOSFETs completely degrade within 5 min or so in deionized (DI) water at room temperature (Figure 6b). The silk substrates dissolve rapidly (∼2 min), followed by a beak down of the array into its individual components. Depending on the dissolution rates of the various constituent materials, each of the individual components then gradually disappears.^12^ SiO_2_ and Si_3_N_4_ have also been employed as multifunctional materials that serve as gate dielectrics, interlayer dielectrics, and encapsulation layers in multiplexed neural sensing arrays.^143^ MgO is also considered a useful inorganic dielectric material that has demonstrated optical transparency, good thermal stability, and high resistivity in numerous applications.^144^

#### 2.2.2 Synthetic Polymer Dielectrics

Considering their commercial availability and ease of processability, a variety of synthetic and elastomeric polymers, including PLA, PVA, PDMS, and PU, have also been widely used as dielectric materials for biodegradable devices.^132,145,146^ The applicability of these polymers as dielectric layers is by virtue of the presence of charged/polar terminal groups, for example, alcohol or acid groups that can be polarized under an electric field.^125^ Since elastomeric polyesters such as PGS more reversibly endure compression than more viscoelastic substitutes, these biodegradable elastomers are also especially useful for transient capacitive sensing applications. A PLGA–PCL composite membrane has also been prepared as an elastomeric dielectric layer for the fabrication of a completely biodegradable pressure sensor using a state of the art electrospinning technique.^147^ PLGA was selected as the main component of the composite to relaize desired dielectric properties (3.3–4.4, depending on molecular weight), while PCL was chosen as a secondary component to realize desired mechanical properties. The nanofibrous dielectric demonstrated sufficient mechanical properties, revealing a modulus of elasticity of less than 10 MPa and attractive degradation characteristics, losing 60% of its initial weight during first 2 weeks of degradation in PBS and completely dissolving within 18 days (Figure 6c). At the same time, after the first week of degradation, the sensor retained 70.5% of its low-pressure measurement range. Because of its highly compressible and porous nature, the PLGA–PCL nanofibrous dielectric presented tunable mechanical and dielectric properties.

On the other hand, synthetic and elastomeric polymers tend to display reduced dielectric constants (κ < 3), which need high voltage for device operation.^148−150^ To realize lower operation voltages, several approaches have been developed for the formation of high-k dielectrics. A general strategy involves the incorporation of inorganic high-κ fillers including SiO_2_ (κ = 3.9), aluminum oxide (Al_2_O_3_) (κ = 9), hafnium oxide (HfO_2_) (κ = 25), titanium dioxide TiO_2_ (κ = 80), and Barium titanate (BaTiO_3_) (κ ≤ 7000) into the polymer matrix. For instance, Al_2_O_3_ additives have been used to form degradable high-k cellulose acetate dielectrics (κ value = 27.57 (50 Hz)).^87,151−153^ In addition, the integration of polymeric materials with conductive fillers, including conductive polymers, carbon nanotubes (CNTs), metal particles, or liquid metals, enhances the effective electrode area and enables polarization, thus resulting in high-k dielectrics.^154−158^ For instance, both metal oxides and CNTs have been shown to improve the dielectric constant of paper, resultant in a high κ value of 3198 (1 kHz).^88^ However, high concentrations of conductive fillers may significantly increase leakage current and adversely affect device performance. Elastomeric polymers integrated with polarizable moieties, such as nitrogen (N), oxygen (O), and fluorine (F), atoms can also be used. For example, the incorporation of highly polarizable PEG units within conventional PU leads to increased dielectric constant.^159^

#### 2.2.3 Naturally Derived Dielectrics

As an alternative, natural polymers also essentially demonstrate innate practical dielectric properties. For instance, due to an abundance of free hydroxyl groups that impart polarity, most plant-based fibers, including cotton, jute, banana, and bamboo, demonstrate high κ values.^160,161^ In addition, cellulose-based gate dielectrics have been widely used in organic TFTs (OTFT) with high on–off ratios.^162,163^ For instance, a high κ dielectric cellulose-based gate dielectric for OTFTs and complementary inverter circuit have previously been shown to out-perform any other organic inverter circuit by demonstrating a record DC gain of above 500 V/V, a low operation voltage of only 4 V, and large noise margin up to 92.5%.^164^ Many protein derived polymers, such as silk, shellac, and gelatin, have also been highlighted as gate dielectric materials for TFT devices. For example, pentacene-based OTFT with solution-processed silk fibroin gate dielectric demonstrated high mobility of 23.2 cm^2^/(V s) and a low operating voltage of −3 V (Figure 6d).^165^ Similarly, shellac and gelatin also display high gate dielectric properties in OTFT.^166,167^

Because of low dielectric losses, high breakdown strength, and low loss tangent, various sugars, such as glucose, lactose, and sucrose, naturally occurring nucleobases, such as adenine, guanine, thymine, and cytosine, and both essential and nonessential nutrients, including caffeine, are also promising dielectrics for biodegradable, biocompatible, and even edible devices.^23,125^ For example, organic FETs (OFETs) fabricated with sugar dielectrics and fullerene-based semiconductors demonstrated capacitances per area of 6.8 nF/cm^2^ and 2.15 nF/cm^2^ for lactose and glucose, respectively, with minimal hysteresis.^168^ Deoxyribonucleic acid (DNA) is another useful biodegradable dielectric that can be the derivative of natural and renewable sources such as waste materials from fishing industries and has recently attracted significant attention for organic electronic and photonic devices.^169−172^ To enhance solution processability for thin-film processing, a complex can be formed between DNA and cationic surfactants such as hexadecyltrimethylammonium chloride (CTMA).^173−176^ Then again, because of the presence of mobile ion impurities, OFETs with DNA-CTMA dielectrics have substantial hysteresis.^174,177^ On the other hand, hysteresis can be reduced by limiting the ionic mobility of DNA-CTMA via poly(phenylisocyanate)-*co*-formaldehyde cross-linking.^174^ Alternatively, 2.5 nm films can be produced from DNA nucleobases using vacuum processing and directly used as biodegradable dielectric materials. For instance, vacuum processed thin films of guanine and cytosine displayed dielectric constants and breakdown voltages similar to both glucose and lactose, while also presenting low losses in the range of 10^–3^ at 100 mHz. Notably, high capacitances per area were also attained, 9.25 nF/cm^2^ for guanine and 13.8 nF/cm^2^ for cytosine.^177^ Egg white or albumen is another interesting nutrient-based dielectric. By simply spin coating and thermally processing albumen obtained directly from eggs, without further extraction, a high-quality dielectric layer in pentacene- and C60-based OTFTs was achieved (Figure 6e).^178^ The dielectric and surface properties of the egg-derived dielectric layer were tailored by regulating the thermal baking conditions and sequences. The thermal processing of the albumen film led to irreversible denaturation of albumen proteins and the creation of the disulfide bonds between two cross-linked protein molecules, which takes a critical part in the decline of gate leakage current.

### 2.3 Semiconductor Materials

#### 2.3.1 Inorganic Semiconductors

**Figure 7:**
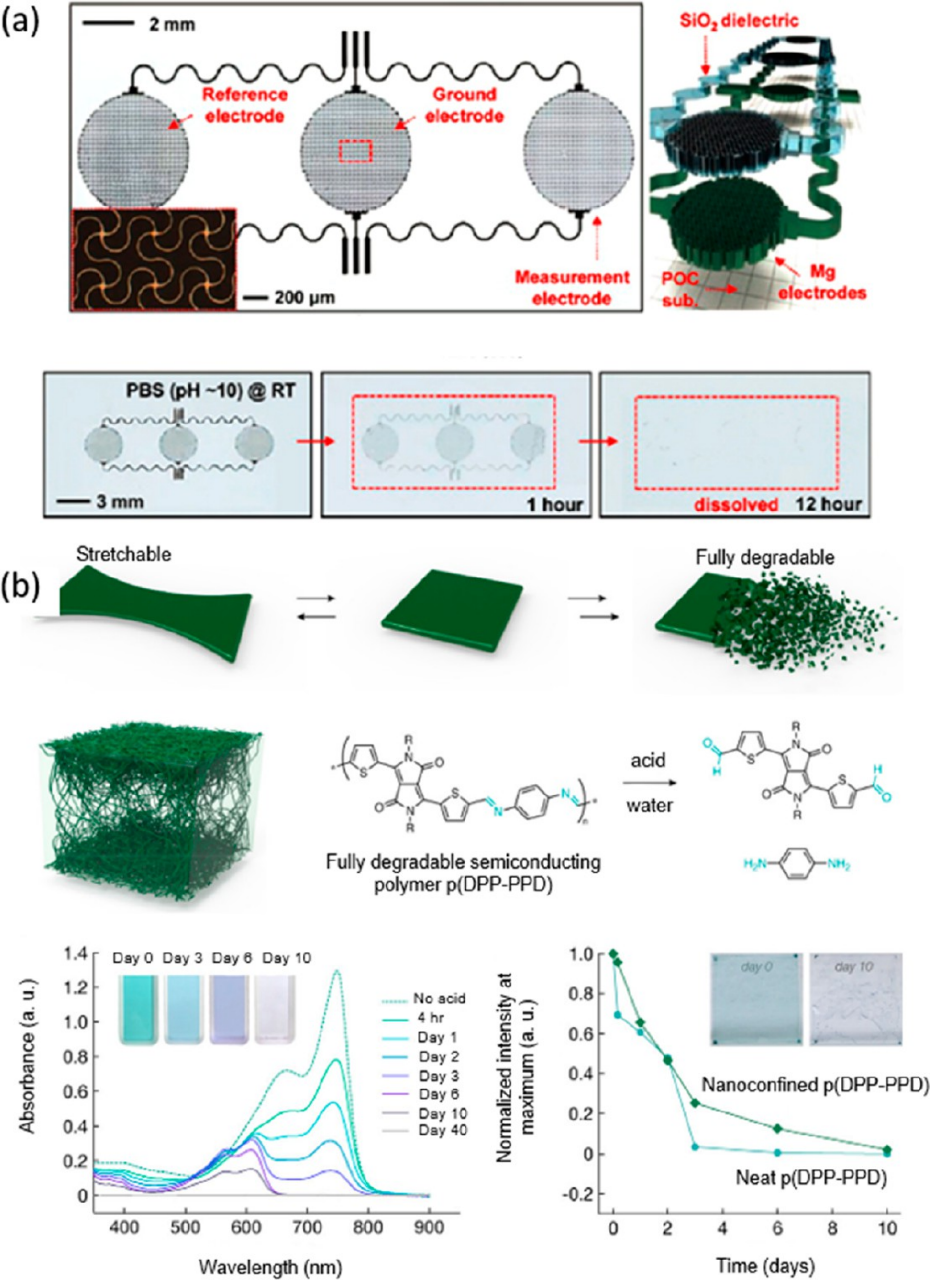
(a) Image of the fabrication and degradation of capacitive electrophysiology (EP) sensor based on biodegradable elastomers and Si nanomembranes/nanoribbons. Reproduced with permission from ref (180). Copyright 2015 American Chemical Society. (b) Illustration and chemical structure of nanoconfined acid-labile semiconductor fibers embedded within a biodegradable elastomer as well as the UV–vis absorption spectra of a solution of p(DPP-PPD) chlorobenzene with the addition of 1% 1 M TFA, and the normalized peak maxima extracted from UV–vis absorption spectra of a thin film of neat and nanoconfined p(DPP-PPD) in 1 M TFA water. Reproduced with permission from ref^207^. Copyright 2019 American Chemical Society.

Traditional semiconductor electronics have been largely dominated by inorganic-based materials such as Si and metal oxides. Since Si is the mainstay material for the semiconductor industry and many technologies, a vast number of fabrication techniques and processing methods have been developed for its deposition and growth.^179^ Furthermore, recent revelations that Si experiences total hydrolysis (relevant only to Si layers in nanoscale dimensions) have also unlocked new opportunities for the use of Si in electriconic systems that require biodegradability.^61^ As a result, a wide range of degradable Si-NM-based devices have been developed. In one example, p- and n-channel metal-oxide-semiconductor field-effect transistors (MOSFETs) have been developed using a biodegradable elastomer POC as a stretchable polymer substrate and Si nanomembranes/nanoribbons to serve as skin conformable, transient, and capacitive electrophysiology (EP) sensor.^180^ This EP sensor demonstrated a linear, elastic-mechanical response, and reversible stretching at strains of up to ∼30%. The transient electronic materials dissolved within hours in a uniform fashion and without delamination, while the POC substrate remains visible for several weeks (Figure 7a). Other alloys of Si, such as SiGe and Si_3_N_4_, have also been explored.^68^ At the same time, growing research surrounding metal oxide semiconductors has resulted in the development of more cost-effective materials, compared to elemental semiconductors, exhibiting comparable device performance and improved ambient stability. Gallium oxide (Ga_2_O_3_), tin oxide (SnO_2_), indium oxide (In_2_O_3_), and ternary oxides such as tin-doped indium oxides (ITO) and fluorine-doped tin oxide (FTO) have all demonstrated comparable performances to Si-based materials.^181−185^ In addition, the end products of the degredation pathway of these oxides are considered unharmful in physiological environments. At the same time, cumbersome processing techniques and a lack of availability make these metal oxides expensive. Instead, ZnO is regarded as a standard metal oxide for various health monitoring devices. The relative ease of synthesis, varied solution processability, stability, and efficient charge transfer properties makes it an ideal material.^186−188^ Besides, the major end product of the degradation pathway is a metabolite processable by the body, zinc hydroxide (Zn(OH)_2_). The biocompatibility and antibacterial properties of TiO_2_ are also particularly attractive.^189^ Nevertheless, to grant a suitable rate of charge transfer, TiO_2_ requires relatively high processing temperatures (∼200 °C) to convert from an amorphous to crystalline phase. Considering biodegradable substrate materials like PLA or PVA may not be compatible with elevated temperatures such as these, transfer strategies may therefore need to be explored.

#### 2.3.2 Synthetic Semiconducting Polymers

The emergence of semiconducting polymers, such as polythiophenes (P3HTs) (e.g., poly(3-hexylthiophene), P3HT)(65), and donor–acceptor copolymers (e.g., diketopyrrolopyrroles, DPP), has revealed a distinct class of materials that offer a more cost-effective solution and greater mechanical flexibility than their inorganic counterparts.^190−193^

Semiconductors are typically characterized by their carrier mobility. In organic systems, charge transfer happens along and between conjugated backbones. The prime feature defining the conductivity of organic semiconductors, or intrinsically conductive polymers, is, therefore, the extent of π-conjugation in the polymer backbone. A longer sequence of alternating C=C double bonds or a higher degree of π-conjugation leads to greater intra- and intermolecular overlap of the π-orbitals through π–π stacking, thus resulting in higher conductivity due to increased delocalization of π-orbital electrons.^194^ While the electrical conductivity and charge transport properties of inorganic materials are still superior, the solution processability and tuneability of π-bonded molecules or conjugated polymers (CPs), including polypyrrole (PPy), polyaniline (PANI), poly thiophene, and poly(3,4-ethylenedioxythiophene) (PEDOT), which has conductivities up to 4.6 × 10^3^ S/cm when doped with poly(styrenesulfonate) (PEDOT:PSS), offer potential advantages over inorganic and small-molecule organic semiconductors.^195−202^

Partially degradable semiconducting and conducting polymers are often achieved by blending nondegradable semiconducting materials or conjugated polymers with biodegradable, insulating polymers. For example, a P3HT derivative with carboxylate substituents known as poly(3-thiophene methyl acetate) (P3TMA) was blended with poly(tetramethylene succinate), PLA, poly(ester urea), and thermoplastic polyurethane (TPU) to enhance miscibility. In this way, although the polymers could not be completely disintergrated into their monomeric constituents, the conductive composites can degenerate. Considering that the electronic component of the polymer blend is often nondegradable, a blend demonstrating maximum electrical conductivity with a minimum concentration of nondegradable conjugated component is most desirable.^23^ As with dielectric materials, conducting polymers can also be achieved by distributing conjugated polymer nanoparticles throughout a biodegradable polymer matrix.^203−206^ To sufficiently form conducting networks within the composite, however, conductive fillers must, unlike biodegradable dielectric materials, be above the critical filler content, also referred to as the percolation threshold. Furthermore, since a high degree of degradability requires a minimum concentration of nanoparticles, this approach is best suited to applications where a lower conductivity is sufficient.^206^

Complete degradation is achieved by introducing hydrolyzable linkages into semiconducting polymer backbones. For instance, a fully degradable, two-component polymeric system has been assembled from semiconducting fibril aggregates nanoconfined in an elastomeric matrix to enable controlled transience and strain-independent transistor mobilities.^207^ Both the urethane-based elastomeric matrix (E-PCL) and the p(DPP-PPD) semiconductor, a diketopyrrolopyrrole (DPP)-based polymer that features imine bonds that were created to completely break down into their monomeric constituents in acidic aqueous solutions. This is achieved by hydrolysis of imine bonds along the polymer backbone. UV–vis absorption spectra, which have been used to monitor the degradation process of neat p(DPP-PPD), in a 1% 1 M trifluoroacetic acid (TFA) (pH ≈ 0.5), chlorobenzene solution show a steady decrease in light absorbance. The light absorbance eventually becomes negligible after 40 days of incubation. The color of the corresponding solution also changes from blue–green to purple and then to clear (Figure 7b). Thin films of both neat and nanoconfined p(DPP-PPD) demonstrate similar but slower dissolution trends in 0.1 M TFA in water, highlighting the potential of these imine-linked semiconductors for transient electronics that will move through and breakdown within the digestive system.^91^ Alternative strategies for complete degradation involve conjugation breaking, whereby flexible but nonconjugated linkers are presented into semiconducting and conducting polymers, also resulting in enhanced processability and mechanical properties, with nominal concession to device activity.^208−211^

#### 2.3.3 Naturally Derived Semiconductors

Naturally available conjugated molecules also offer exciting platforms for biocompatible and biodegradable sensors for futuristic applications. Many conjugated molecules found in dyes and food offer naturally defined degradation pathways and intrinsic biocompatibility. Indigo, a natural plant-derived dye, was one of the earliest reported naturally derived conjugated molecules. Indigo is a semiconductor with a bandgap of 1.7 eV and stable exciton transfer on the order of 10^–2^ cm^2^/V s. Indigo molecules also lack intramolecular π-conjugation but nonetheless display impressive anisotropic charge transfer features. This is due to the strong intermolecular hydrogen-bonding, which underpins π-stacking along the crystallographic *b*-axis.^212^ Naturally inspired synthetic dyes, for example, indigoids, such as tyrian purple, acridones, anthraquinones, terpenoids, phenazines, and perylene/naphthalene imides, have also displayed exciton transfer in the range of 10^–2^–10^–1^ cm^2^ /(V s).^213,214^ The natural pigment melanin is again an interesting biopolymer, which, though essentially nonconducting because of an extremely disordered structure, displays conductivity through doping via water absorption.^215^ Owing to the significance of hydration on melanin conductivity, it is an attractive material for *in vivo* healthcare monitoring applications. For instance, an *in vivo* investigation of fully hydrated melanin thin films with conductivities of 7 × 10^–5^ S/cm revealed that the melanin implants are almost fully eroded and resorbed after 8 weeks.^216^ Other eco-friendly and biodegradable natural conjugated materials include the molecule accountable for the orange color of carrots, β-carotene and byproducts of a natural laxative, anthraquinone.^116,217^ Although these natural or nature-inspired conjugated molecules possess exciting potential, strict processing constraints are required to attain the most advantageous morphology and orientation for sufficient device performance.^218^ Thus, to better understand and predict the future of these materials, a greater assessment of charge transfer and electrochemical features is required.^219^ Similarly, to understand their environmental impact, further in-depth investigation into their mechanisms of dissolution is also necessary.

### 2.4 Electrode Materials

Electrode materials are responsible for transporting electrical charge carriers around electronic or sensing devices and to the external circuit. To achieve high-performance biodegradable devices, conductors with high conductivities (>10^–1^ S/cm) are necessary requirements.^125^

#### 2.4.1 Metal-Based Electrodes

**Figure 8:**
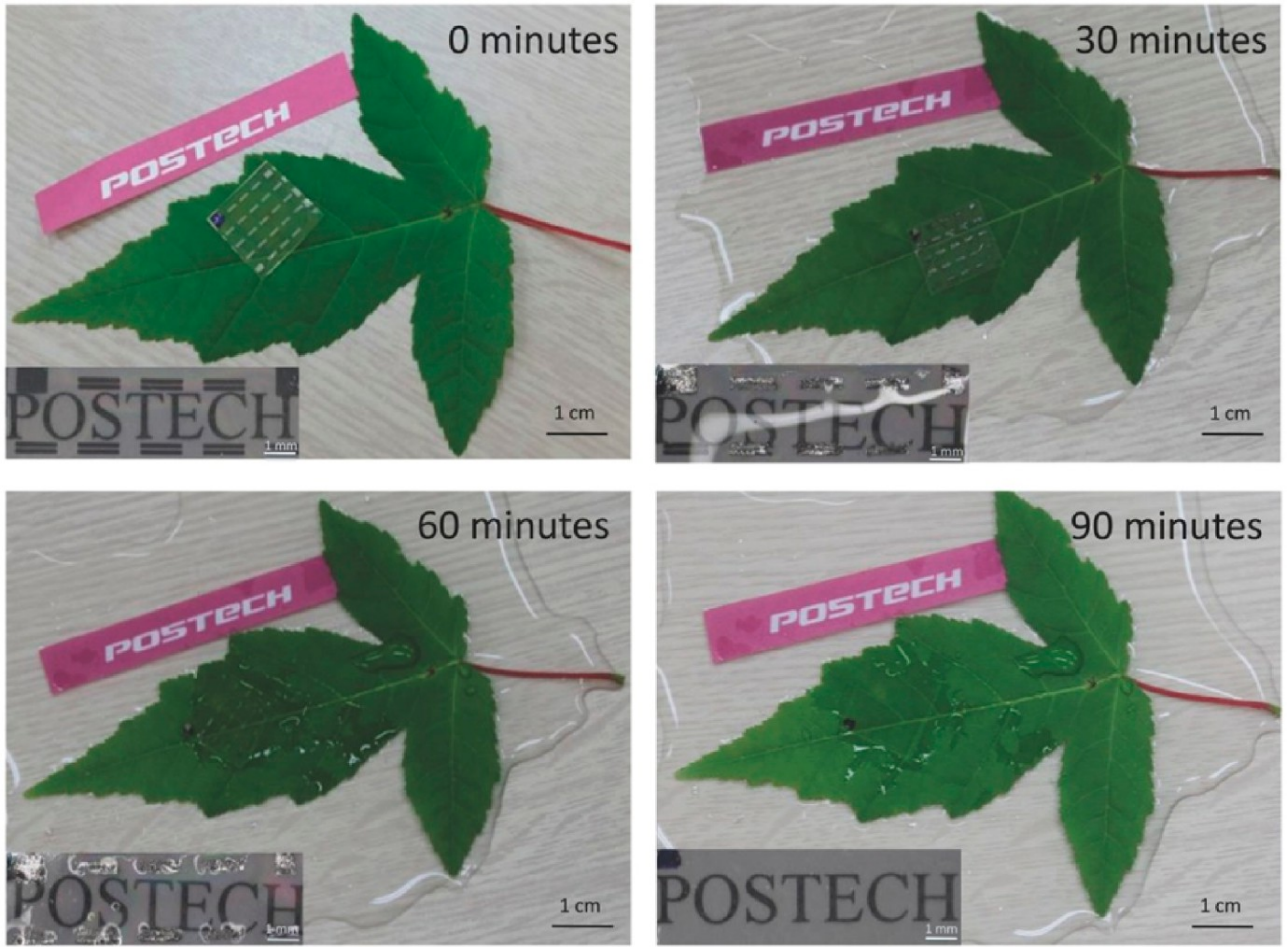
Time-lapse images of the dissolution of Mg electrodes of chitosan-based resistive switching memory devices on a plastic substrate in water at room temperature. Reproduced with permission from ref (221). Copyright 2015 WILEY-VCH Verlag GmbH and Co. KGaA, Weinheim.

Because of their inert nature, corrosion-resistant and high conductivity (σ = 3–60 × 10^6^ S/m) noble metals, such as gold (Au) and silver (Ag), have long been used as electrode materials for electronic medical devices. However, because of their scarcity, these precious metals are usually costly and might result considerable build-up and ultimate obstruction in the body due to their break down resistance. Considering this, the use of corrodible metals linked to trace elements that are intrinsic to the human body, including Mg, Zn, W, Fe, Mo, and their oxides has been indispensible to the early development of biodegradable devices.^62,70,220^ Owing to their ease of processing, lower cost, and safer resorbable properties, Mg and Zn are used most often. For example, Mg has been chosen as an electrode for chitosan-based resistive switching memory devices due to its biodegradability and electrical conductivity (Figure 8).^221^ Nevertheless, both Mg and Zn degrade relatively quickly; metals with slower rates, such as W and Mo, are, thus, more advantageous for devices requiring a long operational lifetime.^70^ In neutral solutions, Fe thin films also demonstrate a slow degradation rate. However, currently available Fe-based materials rust quickly in physiological environments and are subsequently transformed into iron oxides and hydroxides, which feature considerably reduced solubility. The extremely slow degradation of these byproducts can therefore limit the use of Fe-based materials in certain biodegradable health monitoring technologies, particularly edible or implantable technologies that should disappear entirely after fulfilling their requirements.

#### 2.4.2 Carbon-Based Electrodes

It is also worth mentioning that graphite, carbon black nanoparticles, and CNTs have also been employed as organic carbon-based electrodes for several biosensing applications.^222^ Nevertheless, the toxicity of these nanosized materials limits their use in biodegradable devices. Furthermore, because of their enhanced intrinsic charge transfer as well as the ease of processing, metals are largely the prime material of choice for electrodes.

#### 2.4.3 Polymer-Based Electrodes

Organic polymer-based electrode materials are achieving prominence due to their reasonable electronic conductivity after doping as well as their electronic and ionic conduction.^223,224^ In addition, polymers display high mechanical flexibility, in comparison to metal contacts, and are, therefore, more appropriate for the development of flexible and conformal electronic health monitoring devices. Strategies suitable for the preparation of biodegradable semiconducting polymers also apply to conductors. Relevant examples of polymeric conductors are chemically or electrochemically doped conjugated polymers, including melanin, polyacetylene, PEDOT, PANI, PPy, P3HT, and their copolymers. However, due to their lower conductivities, newly emerging fully degradable conducting polymers have mostly been developed to monitor small bioelectronic signals. For the practical realization of fully degradable conducting polymers, better control over the chemistry, morphology, and doping of these materials and much higher conductivities are required.^23^ Another disadvantage of conducting polymer-based electrode is the requirement for orthogonal solvents to circumvent the dissolution of the primary polymer electrode when processing subsequent layers. This may lead to a new or additional challenges that could complicate the device fabrication process further.^125^ Despite these limitations, the development of highly conductive biodegradable polymers will significantly pave to the advancement of bioelectronics interfacing human skin or internal organs. Forthcoming advances in organic conducting materials would, therefore, be among the utmost exciting nevertheless the most essential milestones to attain fully biodegradable high-performance transient devices.

### 2.5 Encapsulants and Adhesives

**Figure 9:**
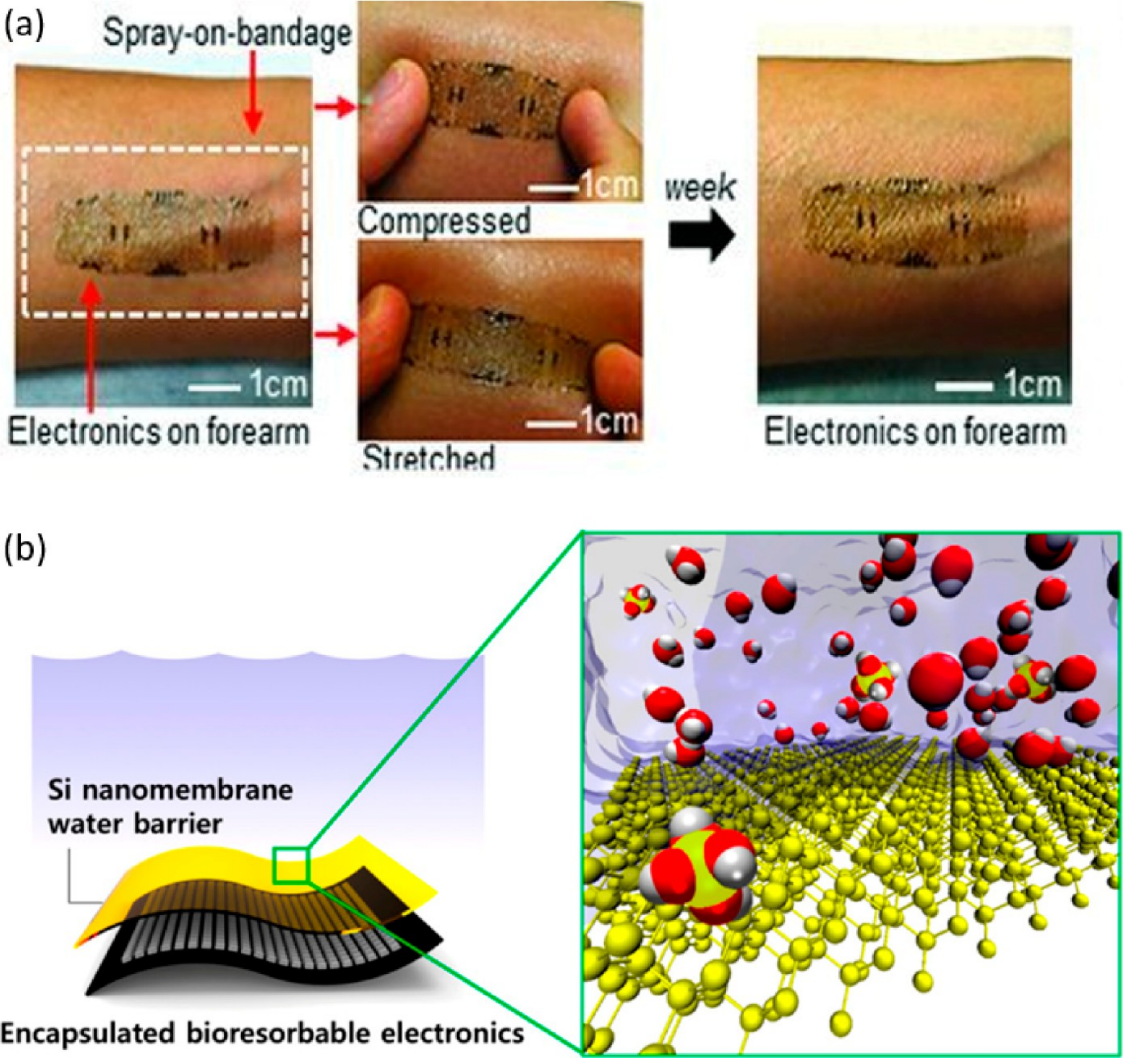
(a) Epidermal electronic systems mounted on a forearm and encapsulated with a layer of spray-on-bandage (left), under compression and extension of the skin (center), and after wearing for 1 week (right). Reproduced with permission from ref (225). Copyright 2013 WILEY-VCH Verlag GmbH and Co. KGaA, Weinheim. (b) Monocrystalline silicon nanomembranes as encapsulation layers for water-soluble electronics. Reproduced with permission from ref (64). Copyright 2017 American Chemical Society.

Like substrates, encapsulation layers also play key roles in defining the lifetimes of biodegradable electronic and sensing devices.^24^ Encapsulation layers provide protection to ensure device performance and safety for its intended lifetime, before the degradation of functional materials, such as organic semiconductors that are generally more susceptible to environmental degradation.^125^ Encapsulants or adhesives also serve as an insulating barrier between different device components that may be built in a multilayered sandwich structure.^125^ Starch and carbohydrate-based sugars, like sucrose, are some of the interesting materials that are mechanically extremely soft to be used as a substrates; however, they could also be used as adhesive materials. Easily dissolved synthetic polymer materials, including PLGA, PVA, POC, PCL, etc., can also serve as a temporary encapsulant for the protection and easy handling of the devices.^225−227^ For instance, epidermal electronic systems (EES) have been developed using water-soluble sheets of PVA that were bonded to and encapsulated on the skin using a spray-on-bandage.^225^ Placement of the EES on the skin was followed by the dissolution of the PVA in water, leaving the EES mounted on the skin (Figure 9a). A polyvinyl acetate (PVAc) encapsulation layer extended the lifetime of pentacene based OTFT devices.^146^ Controlled modulation of the device lifetime has also been achieved by encapsulating devices in multiple air pockets formed using multiple layers of silk.^228^ In wet environments, as the protective silk layer begins to swell the device, the air pockets start to collapse, and the device degradation begins. However, due to solvent incompatibility or discrepancies in surface energy, there will be instances where interfacial adhesive materials are required to promote wettability and interaction. In addition, when a long device lifetime is required the relatively weak resistance of biodegradable polymeric materials to water permeation limit their use. For instance, Mg electrodes encapsulated using silk fibroin lose their conductivity within just a few hours.^12^ Despite this, the biodegradation of encapsulation materials with ultralow water permeation rates still requires investigation. On the other hand, by using Si membranes (∼1.5 m) as encapsulants, the degradation times of dissolvable metals can significantly extend (Figure 9b).^64^ For example, Mg thin films encapsulated by an Si membrane can perform for up to 60 days in PBS solution at 37 °C. SiO_2_ and Si_3_N_4_ have also demonstrated good resistance to water permeation.^143^

## 3 Biodegradable Sensors: Fabrication and Implementation

### 3.1 Physical Sensors

Pressure/strain sensors are one of the most important classes of physical sensors required for monitoring body conditions. Numerous types of biodegradable pressure sensors made up of capacitive structures, that is, those filled with air or biodegradable dielectric materials and piezoelectric, piezocapacitive, or piezoresistive material, have been developed for health monitoring purposes, in addition to electronic skins and soft robotics. They are implanted in different parts of the body to prevent the creation of dangerous intracranial pressure in organs postsurgery in areas such as brain, eyes, or muscles.^1,229,230^

**Figure 10:**
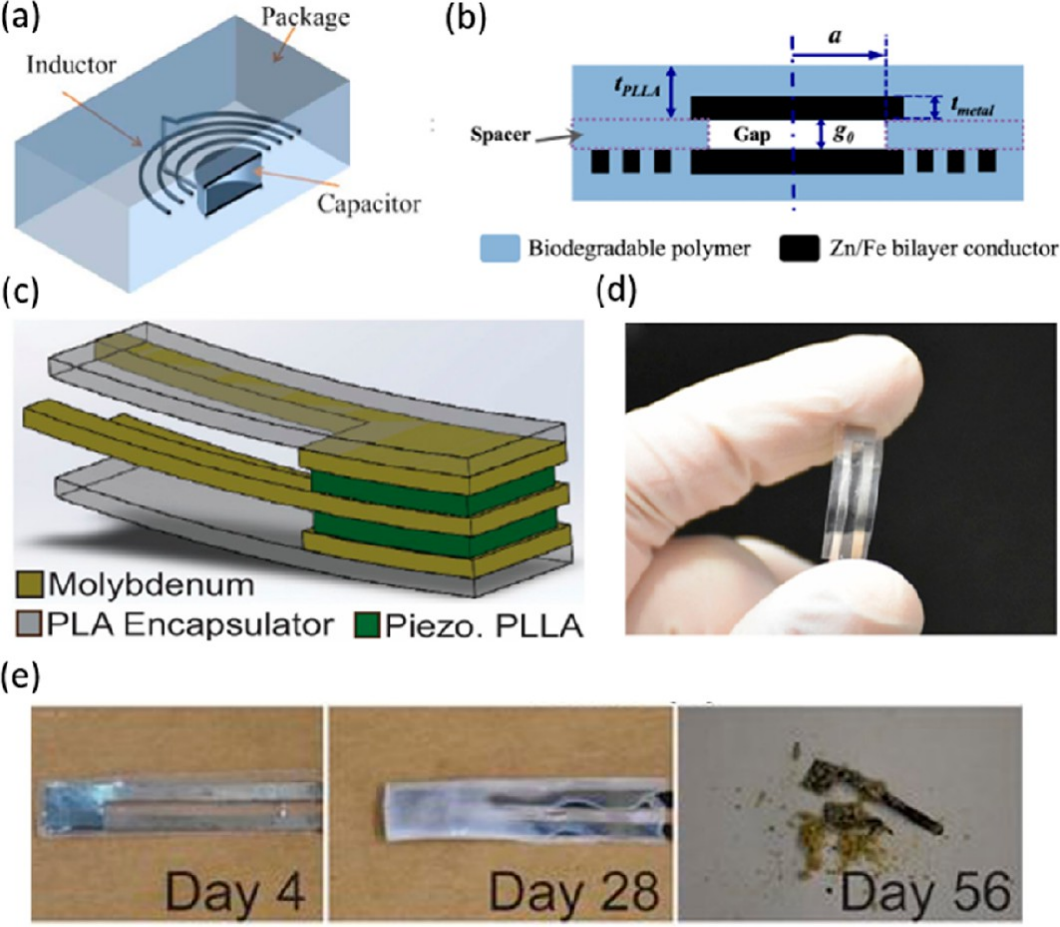
(a) Schematic cross-section of a passive LC resonant sensor and (b) capacitor design. Reproduced with permission from ref (231). Copyright 2014 IEEE. (c, d) Schematic and optical image of the biodegradable piezoelectric PLLA sensor. (e) Sensor degradation at different days in the buffered solution at a temperature of 74 °C. Reproduced with permission from ref (232). Copyright 2018 Proceedings of the National Academy of Sciences of the United States of America.

The examples include a biodegradable wireless capacitive pressure sensor based on the resonant frequency mechanism developed with electrodeposited conductor Zn/Fe bilayers parallel plate, separated by a PLLA layer, and sealed with polycaprolactone (PCL).^231^ The capacitor is filled with air and connected to a microfabricated inductor coil (Figure 10a,b). Applying pressure to the sensor results in the reduction of the gap in capacitive structure and a shift of the resonance frequency of the circuit. The change in the resonance frequency was wirelessly measured with an external coil outside the body. The fabricated sensor demonstrated a linear frequency response with applied pressure, and the sensor sensitivity in the 0–20 kPa pressure range was about 290 kHz kPa^–1^. *In vitro* degradation of the device was tested in saline solution (NaCl 0.9%). Functional lifetime of the sensor in saline solution was about 107 h, followed by complete degradation of the device in 170 h. In another instance, a biodegradable implantable pressure sensor that has a potential to monitor physiological pressure in the brain, lungs, eyes, and heart was fabricated using piezoelectric PLLA.^232^ The PLLA film was heated under stretch to orient polymer chain and induce piezoelectric properties. Later, the film was cut in a specific orientation to maximize piezoelectric response. The sensor consisted of two layers of piezoelectric PLLA polymer between two tiny biodegradable Mo/Mg electrodes and then encapsulating this assembly inside layers of PLA (Figure 10c,d). The device is sensitive enough to detect even very small changes in pressure in the range of 0–18 kPa with two sensitivity values (75 mV kPa^–1^ in the range of 0–2 kPa and 14 mV kPa^–1^ in the range of 3–18 kPa). Its sensitivity can be changed by varying the number of PLLA layers. The sensor has a reliable performance around 4 days *in vitro* (PBS solution at pH 7.4, 37 °C) and *in vivo* implanted in the back of mice. The sensor started degradation after that to self-vanish, and after 8 days there was no detectable signal. However, it took a few weeks to depredate completely (Figure 10e). Hence, tuning the thicknesses of the PLA encapsulated sensor is a highly effective parameter in controlling the degradation time of a sensor. The same group recently developed a biodegradable pressure sensor by using PLLA nanofibers for monitoring of physiological pressures and as a biodegradable ultrasonic transducer for the delivery of drugs across the blood–brain barrier.^233^

**Figure 11:**
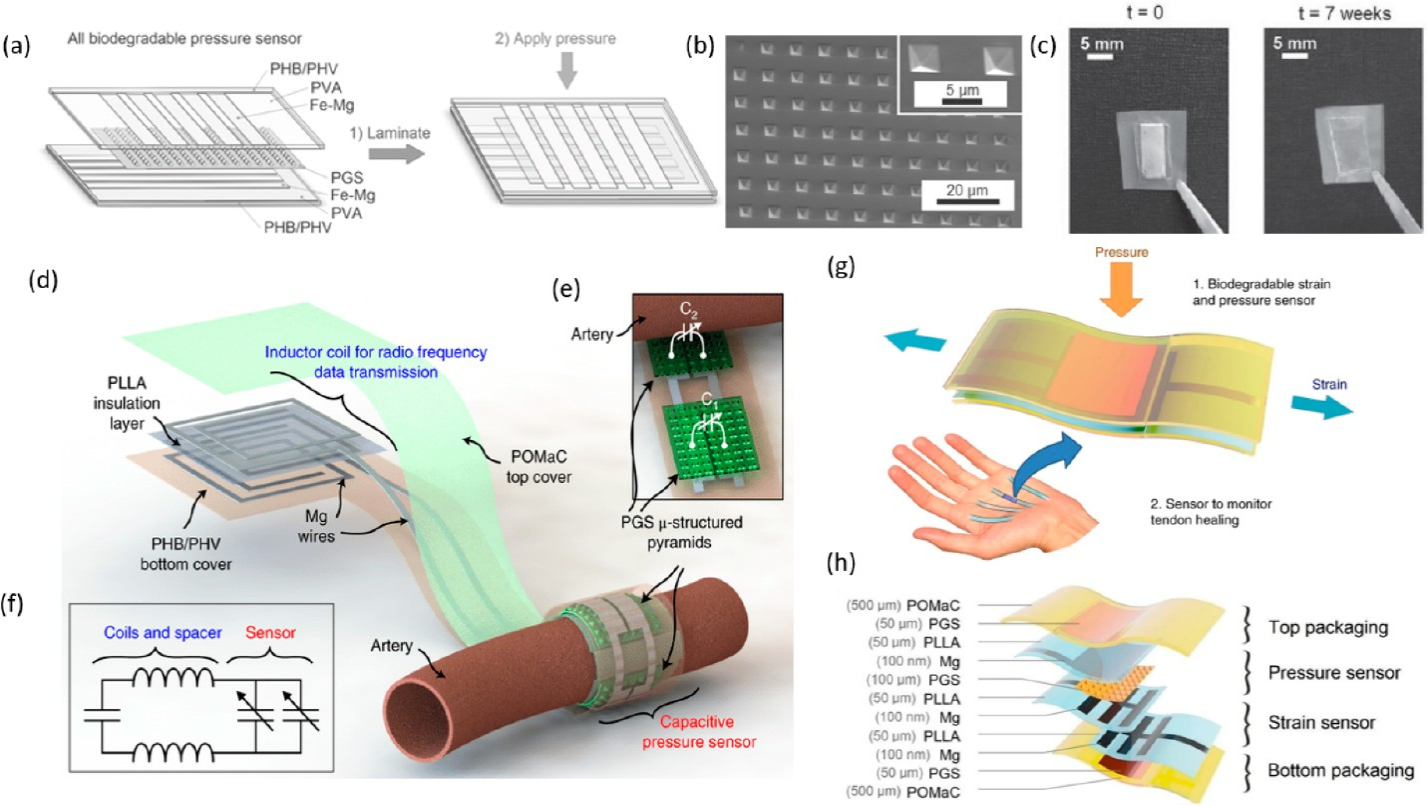
(a) Schematic illustration of the structure of the biodegradable and elastomer PGS-based sensor. (b) SEM image of the PGS film contains microstructured 2D arrays of square-pyramid shapes. (c) Photographs of a device before in vitro degradation and after 7 weeks of incubation. Reproduced with permission from ref (234). Copyright 2015 WILEY-VCH Verlag GmbH and Co. KGaA, Weinheim. (d–f) Sketch of the structure and functional principle of the wirelessly readable capacitive pressure sensor. Reproduced with permission from ref (230). Copyright 2019 The Author(s), under exclusive license to Springer Nature Limited. (g) Implantable strain and pressure sensor that can be attached to a tendon for real-time healing assessment. (h) Materials and overall assembly of the fully biodegradable sensor. Reproduced with permission from ref (137). Copyright 2018 Macmillan Publishers Limited, part of Springer Nature.

The other example of biodegradable pressure sensor is a single-use biodegradable capacitive pressure sensor patch for cardiovascular monitoring using a microstructured dielectric layer of poly(glycerol sebacate) (PGS) between two Mg electrodes as capacitive structure.^234^ Polyhydroxybutyrate/polyhydroxyvalerate (PHB/PHV) were used as top and bottom substrates, Mg electrodes were deposited on them, and finally the PGS layer between them was laminated (Figure 11a). Figure 11b shows SEM image of the PGS film that contains microstructured 2D arrays of square–pyramid shapes. The sensors had a very high sensitivity of 0.76 kPa^–1^ until 2 kPa, and 0.11 kPa^–1^ from 2 to 10 kPa. Blood pulse wave in human arteries was measured successfully by application of the device on the skin, and the skin enhanced the SNR and response time. By submerging the device in PBS at pH 7.4 and 37 °C, Mg electrodes degraded rapidly, and polymers last for a few months as the device retained about 58% of its initial weight after 7 weeks (Figure 11c). The same group recently developed an implantable and self-powered biodegradable sensor based on fringe-field double capacitor structures for measuring arterial blood flow in a healing vessel.^230^ The capacitive sensor had a similar structure to ref (216) (using microstructured PGS dielectric layer and Mg electrodes), and now in this study, a wireless circuit was coupled to an inductor coil to wirelessly track the blood flow. Biodegradable POMaC and PHB/PHV with a thickness of 10 μm were used as substrates and packaging layer and PLLA was used as coil spacer (Figure 11d). The sensor wrapped around the femoral artery of a rat with the softer POMaC layer facing the blood vessel to provide a soft mechanical interface around the vessel wall. The change in capacitance (in pF range) in response to blood-vessels expansion and relaxation generates a shift in the resonant frequency of an LCR circuit that finally read out through coupling with an external reader coil wirelessly (Figure 11e,f). One week after implantation, the sensor signal was decreased. Again, Boutry *et al*.^137^ developed another biodegradable and implantable capacitive sensor for real-time monitoring of the mechanical forces on tendons and tissue strains after surgical repair. The sensor is stretchable and able to measure both strain and pressure of tendon healing at the same time using two independent vertically stacked sensors, as shown in Figure 11g. The sensor consists of microstructured dielectric PGS layer with Mg electrodes as pressure sensor and two interdigitated Mg electrodes that are evaporated on top of PLLA substrate as strain sensor. The entire device is encapsulated in POMaC and PGS (Figure 11h). *In vitro* degradability study carried out by immersing the device in PBS at pH 7.4 and 37 °C showed stable performance for 2–3 weeks. The implanted sensor placed on the back of a rat showed stable signal even after 3.5 weeks. The device has a quite a fast response time (millisecond) and high sensitivity that could discriminate strain as small as 0.4% and the pressure applied by a grain of salt (12 Pa) with no interface between pressure and strain measurements. The fabricated sensor could be used for personalization of rehabilitation protocol.

**Figure 12:**
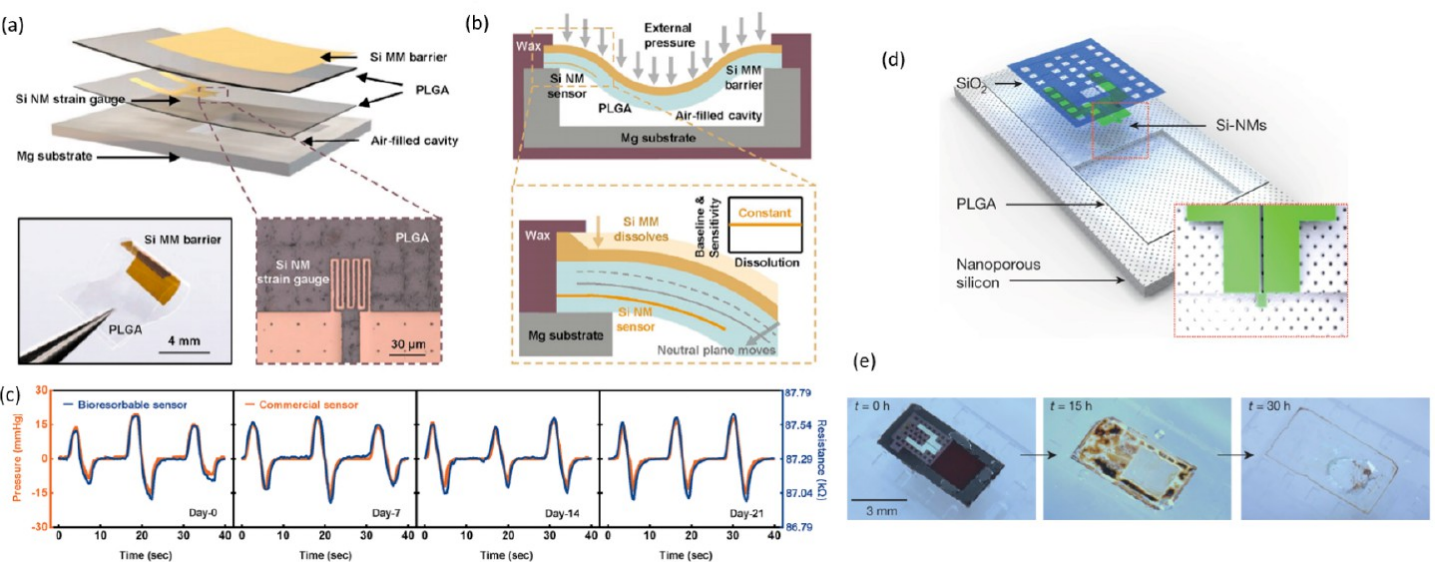
(a) Schematic and optical image of the biodegradable piezoresistive PLLA sensor consists of Si-NM strain gauge as a sensing element. (b) Schematic illustration of the working principle and (c) pressure response performance of the biodegradable sensors (blue line) and its comparison with commercial sensor (orange line). Reproduced with permission from ref (235). Copyright 2020 WILEY-VCH Verlag GmbH and Co. KGaA, Weinheim. (d) Schematic illustration of the piezoresistive silicon-nanomembrane (Si-NM) pressure sensor. The inset shows the magnified illustration of the Si-NM strain gauge. (e) Photographs of the device dissolution upon insertion into an aqueous buffer solution (pH 12) at room temperature. Reproduced with permission from ref (1). Copyright 2016 Nature Publishing Group.

In another report, a bioresorbable pressure monitoring platform with tunable degradation properties for continuous monitoring of intracranial space pressure was investigated.^235^ A Si-NMs-based strain gauge bonded on a layer of PLGA and etched into the surface of Mg with an air-filled cavity in between. The Si-NM offers a piezoresistive response in bending strains due to differences between the pressure of air trapped inside the cavity and the surroundings. The main design feature of the device is that they used a sheet of monocrystalline flexible silicon as a flexible encapsulation layer that is impermeable to biofluids penetration and resorbs in a controllable rate. The device structure was optimized by the theoretical modeling to sensor response, which remains reliable during the partial dissolution of the encapsulation layer (Figure 12a–c).

In a more refined approach, a bioresorbable silicon sensor was developed that can be implanted in the brain and monitor multiple parameters (temperature and pressure) wirelessly.^1^ The device consists of patterned Si-NMs on a 30 μm PLGA membrane with an air cavity and sealed against a supporting substrate of nanoporous silicon or Mg foil (Figure 12d). In response to the pressure of the surrounding fluids, the air cavity allows the membrane to reflect the changes. The sensory response was monitored *in vitro* using artificial cerebrospinal fluid, which displayed the change in resistance of silicon nanomembrane with sensitivity around 0.6 kΩ Pa^–1^ under applying pressure and thermal sensitivity around 0.1 kΩ C^1–^ with increasing temperature. Figure 12e shows the *in vitro* degradation of the resistive pressure sensor in the aqueous buffer solution at pH 12 and room temperature, leading to complete degradation in about 30 h. The device’s functional time could be increased by encapsulating with polyanhydride; however, it decreased the pressure sensor sensitivity to 0.38 kΩ Pa^–1^. The bioresorbable sensor was implanted in the rat brain and wired to an external power supply and communication unit. The sensor could effectively measure intracranial pressure and temperature for 3 and 6 days, respectively. In another recent report, the authors demonstrated the fixation of sensors directly onto orthopedic implants. Mg electrodes were deposited onto the poly(desamino tyrosyl-tyrosine ethyl ester carbonate) (PDTEC) substrates with a holed spacer of polycaprolactone PCL films.

The variable capacitance developed between the electrode changes with the change in the pressure applied, which is detected by the change in the resonance peak. The sensor remained active up to 10 days in Sörensen buffer solution, providing an initial resonance frequency of 101.89 MHz (106.57 MHz in air). The initial resonance frequency observed under immersion in minimum essential medium (MEM) is 92.76 MHz (98.67 MHz in air).^236^

**Figure 13:**
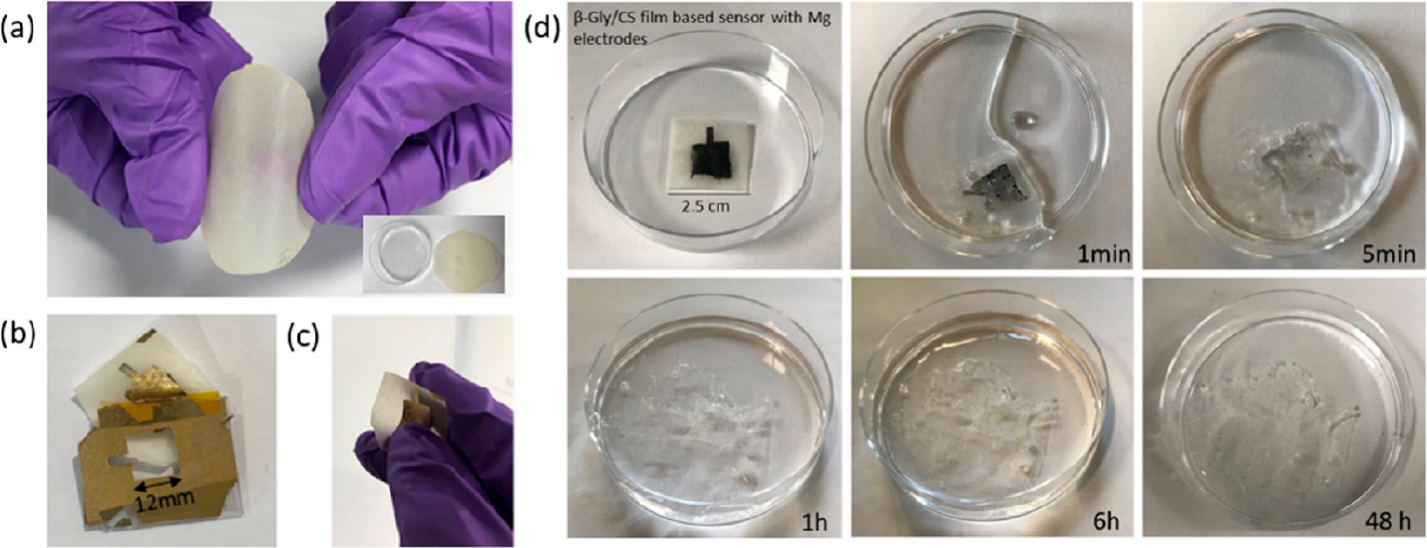
Optical images of the (a) glycine/chitosan (Gly/CS) film and (b, c) deposited Au electrodes on film and fabricated flexible sensor. (d) Dissolution of biodegradable piezoelectric Gly/CS-based pressure sensor with Mg electrodes in PBS solution over time. Reproduced with permission from ref (7). Copyright 2020 American Chemical Society.

In one of our study, a biodegradable piezoelectric pressure sensor based on natural composite of amino acid glycine and chitosan polymer for wearable application was developed for monitoring pressure under the wound bandage (Figure 13).^7^ Deposition of gold electrodes (Ti/Au) of 10/90 nm thickness was done by electron-beam evaporation technique on both the sides of the glycine/chitosan film. The sensor displayed a sensitivity of 2.82 ± 0.2 mV kPa^–1^ under the pressure range of 5 to 60 kPa. The biodegradability of the pressure sensor was studied by depositing Mg as a biodegradable electrode. The electrodes showed complete degradation within a few minutes of immersion in PBS solution of pH 7.4, while the rest of the film dissolved in the next few days. The use of chitosan aided to the flexibility of the film and controlled the crystallization of glycine into β-phase, which is the piezoelectric polymorph of glycine with high piezoelectric coefficient.

A summary of many other bioresorbable sensors, their reported structure, their degradation time, and the *in vitro* or *in vivo* analysis of the samples are discussed in Table 2.

**Table 2:**
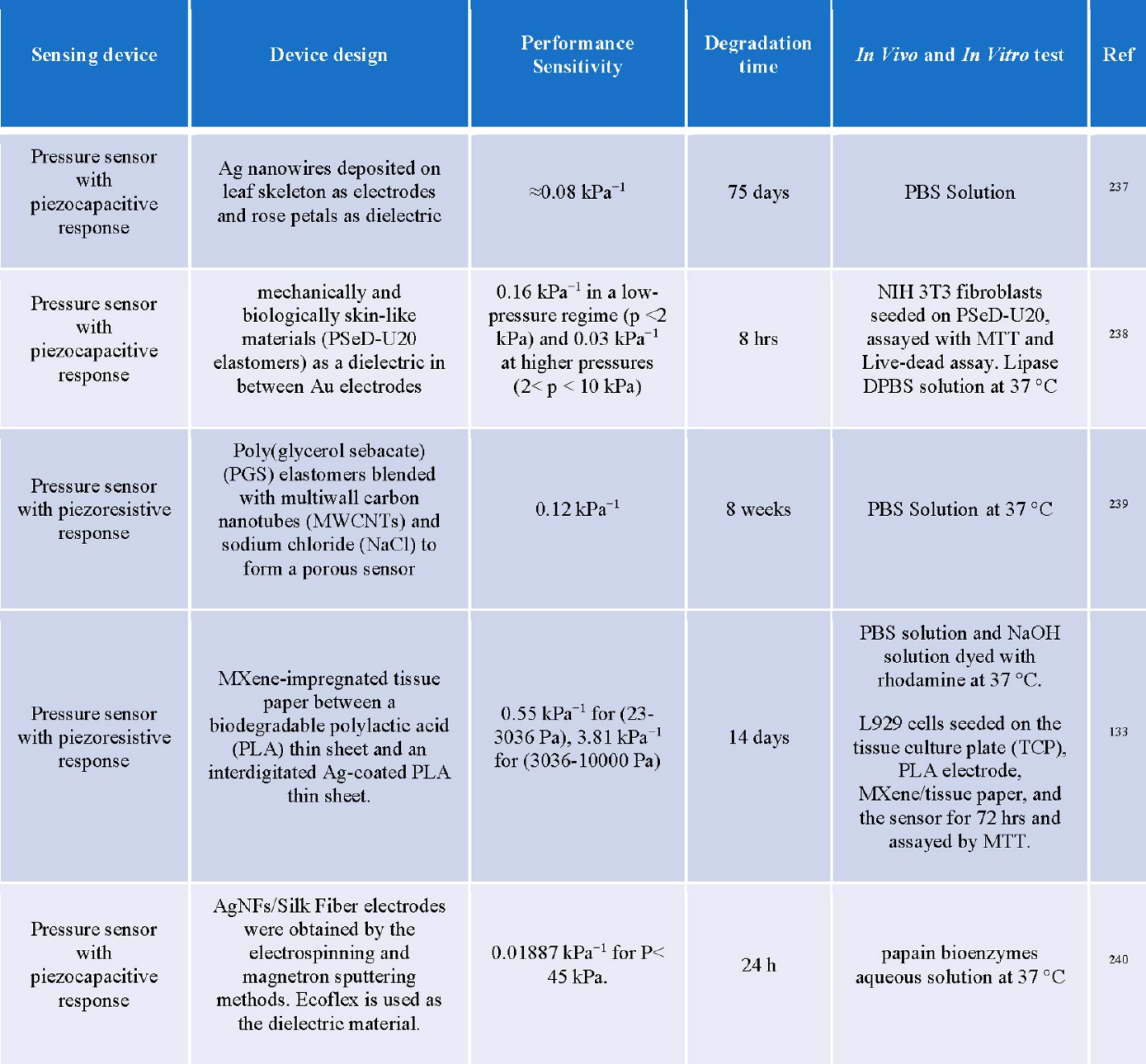
Summarized Glance of Different Types of Biodegradable Pressure Sensors^237,238,239,240^

**Figure 14:**
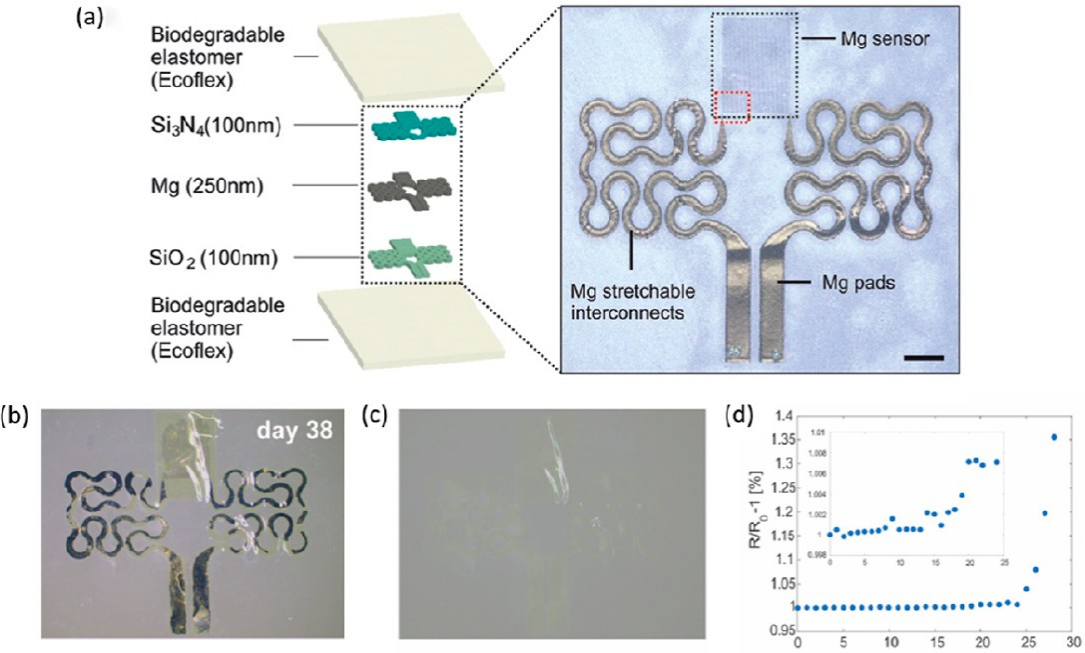
(a) Schematic structure and optical image of resistive temperature sensor consist of metal and semiconductor serpentine structure as sensing element, Peano-like interconnects, and Ecoflex encapsulation. Biodegradability of the sensor in a water–NaCl solution (b) after 36 d and (c) 67 d dissolution in water. (d) Changes in resistance of the sensor after 24 h immersing in the solution. Reproduced with permission from ref (243). Copyright 2017 WILEY-VCH Verlag GmbH and Co. KGaA, Weinheim.

Temperature sensors are also essential physical sensors that provide body temperature measurements for real-time or remote healthcare monitoring and have even been shown to be useful for monitoring intracranial or brain temperature, as well as wound temperature, which can often reflect the incidence of infection.^241^ Temperature sensors are also mainly based on piezoresistive materials. The dependence of resistance on temperature is an interesting feature exploited to build up temperature sensors. Materials developed for sensors display two kinds of nature: their resistance increases as temperature increases (positive temperature coefficient, PTC) or decreases as temperature increases (negative temperature coefficient, NTC). Hence, sensors with PTC behavior could be utilized for overcurrent protection materials, self-regulating heaters, and microswitch sensors. However, NTC behavior material could be utilized for highly stretchable thermistors for wearable temperature sensing, mapping, and compensation applications.^242^ In another report, the authors presented a fully biodegradable and highly formable resistive temperature sensor for medical postsurgery monitoring.^243^ Mg serpentine structures that sandwiched between two dielectric layers of Si_3_N_4_ (100 nm) and SiO_2_ (100 nm) are fabricated on a substrate by common lithography techniques and transfer printed on the compostable flexible Ecoflex polymer (Figure 14). The device is encapsulated by ultrathin films of biodegradable Ecoflex films (<20 μm), which exhibits small swelling rate and elasticity similar to muscles and cartilage. The device exhibited a linear behavior in the temperature range of 20–50 °C and with absolute sensitivity of about 70 Ω K^–1^. The degradability of the sensor was tested *in vitro* using saline solution at 25 °C, which showed a stable temperature measurement for 1 day and that full dissolution of the SiO_2_/Mg/SiN_3_ occurred within 67 days. The fabricated sensor is scalable by integration of an array of sensors to perform a 2D mapping of the temperature distribution.

**Figure 15:**
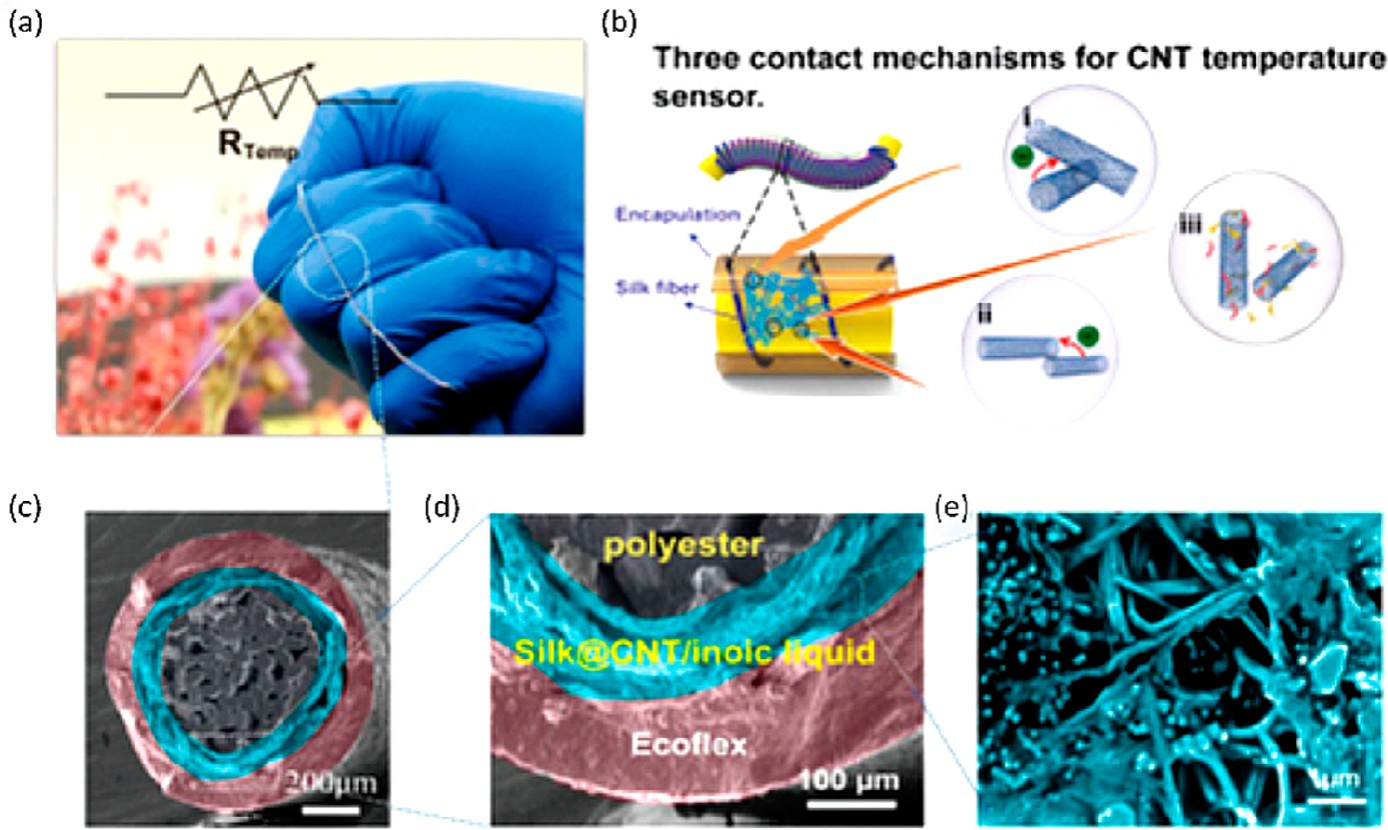
Composite temperature sensors using different sensing materials. (a) Image of a fibrous temperature sensor. (b) Three different conducting mechanisms for composite CNT temperature sensors: (i–iii) Three different electron/ions transport modes in CNT and [EMIM]Tf2N composite sensing materials: (i) transport of charges via the body–body contacts among CNTs; (ii) transport via the end to end contacts of CNTs; (iii) transport via ionic liquids. (c, d) Scanning electron microscopic (SEM) images of the cross-section of a fibrous temperature sensor in different scales: Ecoflex constitutes the outside sealing layer, silk fibers encapsulated with CNTs and [EMIM]Tf_2_N turn out to be the middle layer, and polyester fibers were chosen as the supporting cores. (e) SEM displays the surface morphology of composite material of CNTs and [EMIM]Tf_2_N between the middle silk coiling fibers. Reproduced with permission from ref (244). Copyright 2019 WILEY-VCH Verlag GmbH and Co. KGaA, Weinheim.

In another study, the authors reported the functionalization of silkworm fiber coiled yarns with a mixture of CNTs and an ionic liquid ([EMIM] Tf_2_N). Later, the yarns are coated by highly elastic and biocompatible Ecoflex as the encapsulation layer for the temperature sensor. It showed a temperature sensitivity of 1.23% °C^1–^. The authors explained the increased conductivity displayed to the transport of charges through direct body contacts of the CNT tubes. Another route is via the end to end contact of the tubes. The use of ionic liquid functionalization enhanced the temperature sensitivity and decreased the overall resistivity compared to the only CNT coated yarns, as the ionic liquid provides a site for charge carrier transmission, even at sites with no direct contacts of CNT tubes in the composite (Figure 15).^244^

**Figure 16:**
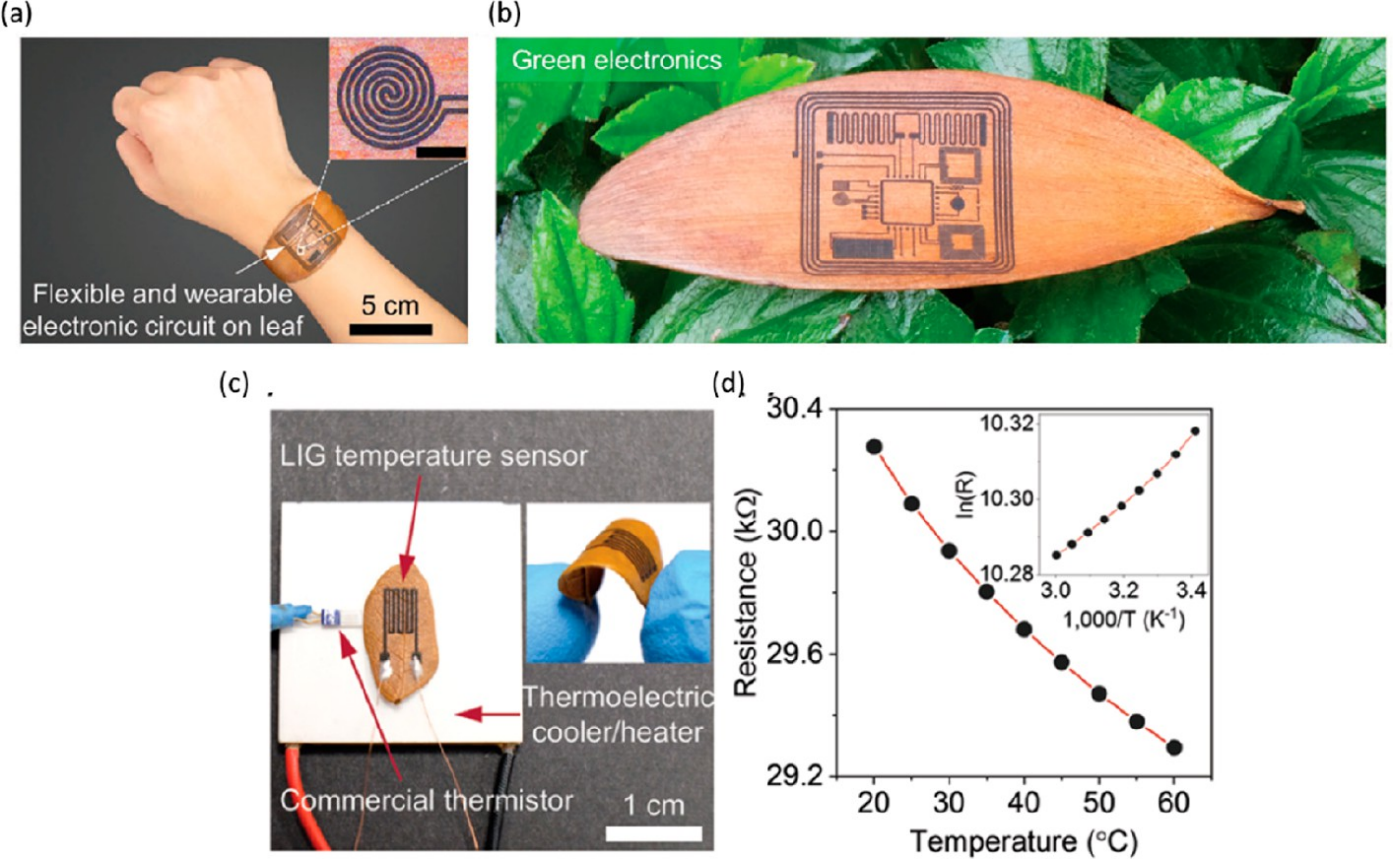
(a) Photo of an LIG electronic circuit on a thin leaf for flexible and wearable devices. The inset shows an enlarged optical image of the temperature sensor (scale bar: 1 mm). (b) Photo of LIG electronics on a leaf for green electronics. (c) Photo of the LIG temperature sensor on the leaf and an experimental setup for measuring performance. The inset shows the flexibility of the leaf that was still retained after FsLDW. (d) Resistance variation as a function of temperature, indicating a negative temperature coefficient behavior. The inset shows the dependency of ln(*R*) on 1/*T*. Reproduced with permission from ref (245). Copyright 2019 WILEY-VCH Verlag GmbH and Co. KGaA, Weinheim.

In another report, intending to produce green electronics, a femtosecond laser direct writing (FsLDW) of graphene patterns on arbitrary natural woods and leaves was reported. The authors used UV femtosecond lasers to transform high power source laser to char wood components by localizing the heat generated (Figure 16a,b). Further, the repeated rate of carbonization resulted in laser-induced graphene pattern creation. The temperature sensor fabricated on a dried leaf (≈ 100 μm thick) displayed a negative temperature coefficient (−0.08% °C^1–^) as shown in Figure 16c and d. They also fabricated a sensor over wood, which displayed a similar result to the leaf, but with longer response and recovery time than the latter.^245^

**Figure 17:**
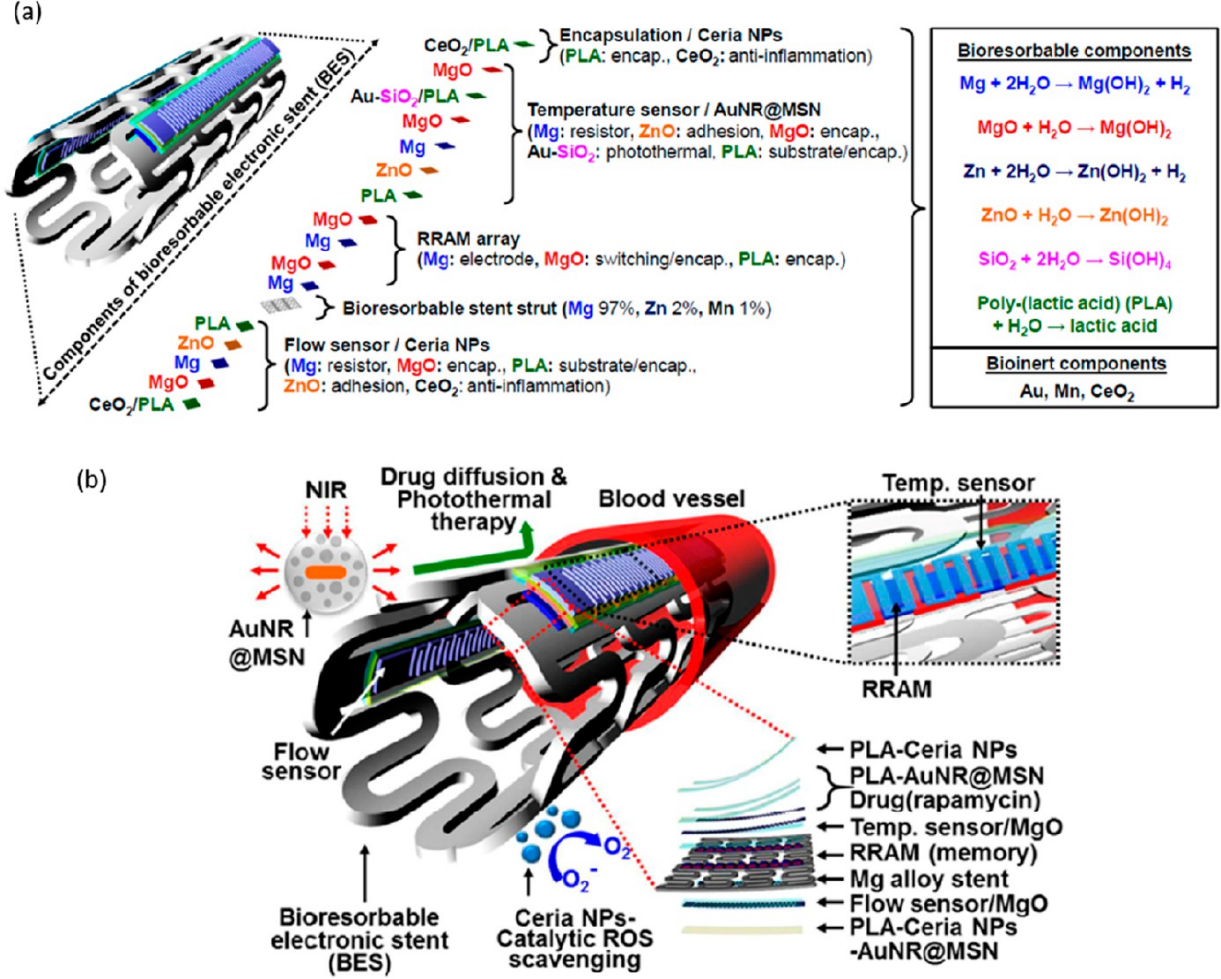
(a) Components of a bioresorbable electronic stent (BES) and bioinert materials and (b) schematic diagram of the BES that includes Mg alloy stent integrated with ceria NPs (catalytic ROS scavenging), AuNR@MSN (photothermal therapy), drugs (e.g., Rapamycin; well-known drug for the treatment of restenosis), flow/temperature sensors (physiological signal sensing), and RRAM array (data storage). Reproduced with permission from ref (246). Copyright 2015 American Chemical Society.

In another study, a nanomaterial was designed for a bioresorbable electronic stent (BES) with drug-infused functionalized nanoparticles. It is utilized to detect flow sensing, temperature monitoring, data storage, wireless power/data transmission, inflammation suppression, localized drug delivery, and hyperthermia therapy. Figure 17 provides a more summarized view of the biodegradable electronic stent developed. The role of ceria nanoparticles was evaluated by both *in vitro* and *in vivo* cytotoxicological analysis. The human umbilical vein endothelial cells (HUVECs) exposed with reactive oxygen species showed a decreased viability. In contrast to the cell lines in the presence of ceria, nanoparticles showed increased proliferation. The *in vivo* analysis by placing the BES in canine common carotid artery displays lower inflammatory responses and macrophage migration.^246^

In an another context, the authors report the development of a bioresorbable photonic device utilized to measure the activity of tissue oxygenation, temperature, and neural activity. The device is made up of several bioresorbable optical components along with single-junction photodetectors, which is based on nanomembranes of device-grade monocrystalline silicon (Si nanomembranes), foundry-produced tricolor photodetectors based on trilayer stacks of Si P–N junctions, an optical multilayer filter of SiOx and SiNy, and optical fibers made up of poly(lactic-*co*-glycolic acid) (PLGA). The concentrations of Si and Zn were measured for 7 days in the blood, brain, heart, kidney, liver, lung, muscle, and spleen tissues of mice using inductively coupled plasma optical emission spectrometry (ICP-OES) and inductively coupled plasma mass spectrometry (ICP-MS). The concentration of the elements increased for the first few days in the kidney, which indicated the renal clearance of the elements.^247^ The photodetector completely degraded completely within 2 weeks of being immersed in PBS solution at 37 °C. The temperature-dependent resistance of the Si nanomembrane photodetector enables researchers to monitor the change in tissue temperature at a resolution of ∼0.1 °C.^247^

An optical sensor for intracranial pressure and temperature monitoring was reported recently. The sensor is designed to monitor the changes in the thickness of an air cavity or in the lattice parameters of the photonic crystal for pressure-induced deflections of Si-NM diaphragm. This causes a shift in the position of resonant peak in the reflection spectra. The same configuration could be adopted to detect temperature change as by monitoring the refractive index of silicon. The temperature responsivity was estimated to be 0.090 nm/°C and pressure sensitivity of 3.8 nm/mmHg. Complete dissolution was observed within ∼195 days in PBS solution at 37 °C.^241^ Shin and co-workers reported the fabrication of an inorganic bioresorbable pressure sensor based on four silicon nanomembrane sensors. Two of which served as strain gauges, and two acted as temperature gauges. The pressure sensor was made up of strain gauges filled with air cavity. The piezoresistive response of the pressure sensor was cast due to the change in the pressure between the surrounding and the air present inside the cavity. The temperature gauges remain unresponsive with the pressure alteration as they are not located on the diaphragm, while they display changes in the surrounding temperature via their temperature-dependent resistance. The pressure sensitivity was estimated to be −0.13 Ωmm Hg^–1^ while the membrane thickness was reduced as well as the thickness of the diaphragm was increased. Similarly, the temperature sensitivity of the sensor was observed to be 0.0012 °C^1–^. The concentration of Si was measured for 7 days in the liver, spleen, heart, kidney, brain, and lung tissues of mice using ICP-OES. The results show higher concentrations of silicon in the spleen, heart, and lungs in comparison to the brain where the device is located. The cerebrospinal fluid transports through the brain and then joins the bloodstream by reabsorption. The phagocytic cells of the spleen and lungs easily uptake these nanomaterials, resulting in the high deposition of Si in these organs. Complete dissolution of the device was observed within a few hours in PBS solution at 95 °C.^229^

There have been several reports on bioresorbable temperature and pressure sensors as part of the medical implants discussed in the last section. They reduce the risk of infection as they limit the need for postsurgical removal. The current impasse lies on the fabrication of biocompatible sensors at a miniaturized scale. The choice of biopolymers is critical, as often they tend to face the issue of thermal lability. Similarly, the soft materials tend to display low Young’s moduli, which cause issues during implantation. Thus, clinical implementation is still a distant goal that requires extensive investigation in animal models and then consecutively in humans.

### 3.2 Chemical Sensors

Chemical sensing devices that can quickly, conveniently, and noninvasively collect information accessible from biomarkers found in the human body, such as glucose, pH, nitric oxide and ions,^15^ are also necessary for the real-time and continuous monitoring of an individual’s dynamic health status.^6,11^ Although various biodegradable physical sensors with comparable performance to their nondegradable counterparts have been demonstrated, there are limited reports detailing the development and performance of biodegradable chemical sensors that continuously monitor biochemical markers for healthcare applications. Thus far, biodegradable chemical sensors have mainly been used for the detection of ions or small organic molecules, possibly due to the challenge of sensing larger and more complex biomolecules *in vivo*. The main challenges surrounding the development of biodegradable chemical sensors relate to their operational lifetime when in direct contact with physiological environments without packaging and the precise measurement of target chemical without interference by other chemicals in biological systems.

A mesoporous chitosan-based conformable and resorbable biostrip for dopamine detection was recently reported.^8^ The electrochemical sensor uses bioresorbable materials such as chitosan, rGo, and graphene, which makes it useful for *in vivo* applications. Furthermore, it improves the detection limit up to 10 pM. In another study, the authors developed a biocompatible and biodegradable polysaccharide-based flexible humidity sensor for wearable applications such as monitoring human breathing states.^248^ A 9:1 chitosan to lignin mixed solution was spin-coated on a flexible PI substrate to yield a sensing layer film with a thickness of approximately 20 μm, and Mg/Fe electrodes were deposited on top of the film. The functionalized chitosan film gives a high humidity sensitivity by the formation of hydrogen bond networks between water and polysaccharides, which accelerates proton transfer under humid conditions and thus increases conductivity of the material. The biocompatibility of the device was confirmed by cytotoxicity tests using human skin fibroblast cells and human umbilical vein endothelial cells in contact with the biocomposite film. In a similar attempt, Liu et al. reportedly used spider egg sac silk (SESS) as a section of single-mode fiber (SMF) to configure an interference-cavity structure. The change of ambient humidity results in the change of SESS diameter, which changes the length of the interference-cavity. The interference spectrum displays a redshift, with the change of the interference-cavity length, and consequentially, the SESS-based sensor displays the humidity-sensing characteristic. The testing results show that the higher is the ambient humidity, the higher is the sensitivity. The maximum value of the sensitivity is 0.99 nm/%RH in the humidity range of 90–99%.^249^ The use of spider silk as potential humidity sensor has been previously explored.^250^

Nanocomposites of zein with graphene oxide (Z-GO), Laponite, and CNT were fabricated using drop casting technique and tested for fabricating for the detection of gliadin. Gliadin is a protein molecule responsible for several autoimmune disorders. The pristine zein coated electrode did not display any electrochemical sensing, while the three composite structures improved the detection response considerably. The CNT-based composite showcased the strongest signals compared to other nanomaterials. The CNT-based composite aided as a natural linker for several functional groups, which ensured a higher degree of performance. The limit of detection observed for these sensors was as low as 0.5 ppm.^251^

## 4 Challenges and Constraints

Despite great progress in the fabrication of biodegradable and implantable healthcare monitoring devices, the high-performance devices that can compete with more established nondegradable healthcare monitoring technologies remain a significant challenging, and many of the sensors discussed above are not yet mature enough for commercialization. For instance, for the clinical implementation of biodegradable healthcare monitoring, devices require reliable and stable electrical performances, high mechanical deformation similar to biological systems, and full biodegradability. In addition, the need for biodegradable power sources culpable to run for a definite time period also remains largely unfulfilled.^252^ There are many other aspects of biodegradable devices that must also be considered before the realization of clinical healthcare monitoring, which have been discussed below.

### 4.1 Degradation Kinetics and Biocompatibility

Degradation kinetics of bioresorbable materials and a functional lifetime of the devices in more complex biological environments need to be investigated carefully as the biodegradation rate in physiological temperature (37 °C and may increase up to 41 °C) and under mechanical stresses due to movements and deformations of body is higher than in a normal lab environment. The physio-chemical parameters (e.g., pH value, oxygen level, and ion concentrations) are also affected by tissue area and may change in a different part of the body. Currently developed wireless biodegradable sensors often have short lifetime for clinical application, and applying proper packaging is an important step to achieve controllable degradation rate. A significant challenge is that biofluid usually penetrates the packaging layers that are based on biodegradable polymers (such as silk fibroin, collagen, and PLGA) and does not provide fully controllable degradation.

Additionally, biochemical interactions between the implant and body environment and cytocompatibility of the degradation products should be specified by appropriate *in vivo* device characterization. Distribution of dissolved biodegradable materials in the body and daily allowance of the materials of the sensor that can be harmlessly resorbed or expelled by the body need to be determined carefully by clinical studies and in consultation with the United States FDA.

### 4.2 Power Supply

Low energy consumption and appropriate device size are of the main constraints in developing biodegradable sensors for clinical applications. Previously developed biodegradable sensors are mostly passive and do not require an internal power supply; therefore, their functionality is limited.^253^ Bioresorbable power supplies, either batteries, energy harvesters, or flexible degradable circuits for wireless energy transfer, are essential components to achieve a full active electrical system in implantable biodegradable devices. Biodegradable batteries using biodegradable Mg as anode materials, Mo as cathode materials, and electrodes space filled by PBS solution have been reported.^254^ To increase the output voltage of the battery, the cells can be stacked in series and a layer of polyanhydride used a separator between cells and packaging. The battery reported using this approach is fully biodegradable, and stacked cells could provide a voltage up to 1.6 V. Another example of biodegradable battery uses Mg and Fe as electrodes and employed similar to biological fluids solution such as MgCl_2_, PBS, and NaCl as electrolytes that could provide an average power of 30 μW for 100 h.^255^ PLC polymer encapsulates the device and is used as a separator between the electrodes. Batteries can provide stable output voltage and high energy density. However, in both mentioned cases, immersing the batteries in biofluid solution at 37 °C showed a limited lifetime of only few hours to a few days and started degradation afterward. In context with energy storage devices, the use of sweat or sweat equivalent solutions as electrolyte is a notable advance.^25^

Degradable energy harvester devices are the other possibility to power the sensors, particularly for the devices that need low power consumption and a long-term operating.^256^ In this regard, piezoelectric and triboelectric generators are ideal for *in vivo* energy harvesting as they produce energy from mechanical motion of the body. A biodegradable piezoelectric harvester by deposition piezoelectric zinc oxide strips on a silk substrate and using Mg as electrodes was reported.^72^ However, the silk substrate quickly dissolved in PBS and lost its functionality; therefore, encapsulation is needed for implantable applications. An example of biodegradable triboelectric nanogenerator includes using multilayered nanopatterned PLGA and PCL film and Mg electrodes to generate the open-circuit voltage up to 40 V.^257^ Other notable advanced solutions related to energy generation reported recently are sweat-based biofuel cells.^258,259^ The main drawback in all of the mentioned energy devices is that they are bulky when compared to the sensor itself and usually lose their functionality soon when operated in biofluids, and they are usually not flexible enough. In addition, for piezoelectric/triboelectric harvesters, their output power under application of a periodic compression force would be enough for sensors operation, but their performance depends on motion or pressure of the body part to which they are implanted.

The other current possibility for implantable sensor power supplies is wireless power transfer.^260^ Both power and data can be transmitted wirelessly to the implant via different wireless transfer techniques, namely inductive, microwave, capacitive, acoustic, and optical. Wireless operation increases the complexity of the system and increases the overall size of the implant but eliminates the risks of infection at the skin and wire interface. Currently, power transfer to the implantable medical devices is typically through inductive coupling technique.^261,231^ The main limiting factors for wireless implants in inductive coupling are related to miniaturizing the size of the coils/antennas used for wireless communication. In most cases, the dimensions of the antenna can not be altered as they depend on the transmission frequency, which depends on the location of the implant in the body and the layers of tissue involved in transmission.^262^ In many cases, the living cells would be absorbing a high amount of transferred energy in the body environment. Research on the other techniques of wireless power transmission such as acoustic^263^ and optical^264^ provided the possibility of miniaturized transmission coil for implants. Nevertheless, having these systems with biodegradable properties has not been explored much.

### 4.3 Data Communication

Data communication in biodegradable sensors is simply achieved by thin wires connected to an external circuitry and power supply, etc.^232,265^ In the case of biomedical applications, the wires could also increase the risk of infection and limit the mobility. In some biodegradable sensors with implanted wireless data transmission, circuitry for wireless data communication has included nonbiodegradable components that would lead to a partially biodegradable system.^1,233^ In this scenario, the nondegradable electronic circuits is usually implanted just under the skin and connected via biodegradable wires to the sensors that are implanted deeper in the tissues. In such cases, the nondegradable circuitry could be removed with a minor treatment after the functional lifetime of the sensors is over and they are left to safely degrade within the body.

Some biodegradable sensors integrated with fully transient wireless technologies have been used in the implanted devices to wirelessly communicate with the external circuitry to enhance the mobility and reduce the risk of infection.^234,266^ The majority of them are based on passive wireless data transfer through resonant inductive coupling between the implanted device and external circuitry.^230,253^ In passive systems, the implanted coil is connected to a sensor, which is usually a capacitive sensor. The change in the parameters such as pressure or temperature results in a change in the capacitance of the sensors and causes a change in the resonance frequency of implanted coil, and information is then transferred to an external reader coil by inductive coupling. The resonance frequency of external coil can be recorded with an impedance analyzer. Passive inductive systems have simple structure, low weight, and enable battery free operation. However, these devices work only for short distance communication (few milimeters), and measured signal strongly depends on the intermediate tissue and position of the coil.^267^ In addition, the sensed signals are mainly limited to a shift in the position of resonant frequency of the external coil, and the operation is limited to short frequency range.

The continuous monitoring of biomarkers using biodegradable wearable sensors could generates large data, which may also require complex circuits for processing. The fabrication a fully biodegradable active electronic circuit with multiple functionalities is a challenging task, and a suitable solution is needed to manage the large data. A few examples in this regard include a biodegradable circuit involving various components such as transistors, resistors, diodes, inductors, capacitors with interconnects developed using conductive Mg, Si-NMs semiconductor, and MgO (or SiO_2_) dielectric layer on a silk substrate.^12^ The transient circuit integrated with degradable sensor, power supply, and wireless control system was implanted in the mice as a fully biodegradable system. Another example of active bioresorbable electronic circuits has been reported for wireless power harvesting and radio communication capabilities.^268^ The functional biodegradable circuits such as those mentioned earlier increase the complexity of the system and the size and power consumption, which is undesirable for implanting. To minimize the implanted circuit size and reduce power consumption of the system, processing of complex data can also be performed outside the body.^269^ For partially biodegradable wireless sensor, another way of power reduction is to focus on antenna modules and radio frequency (RF) components. The choice of wireless technology such as wifi, Bluetooth, or radio frequency identification (RFID) impacts the choice of electronics for the sensor node.^270^ In recent years, the development of the RFID chip has improved the power consumption and its communication range. For instance, the RFID chip sensitivity and read range have improved for far-field communications (FFC) from −8 dBm with a reading range of 5 m in 1997 to −22 dBm with a reading range of 25 m in 2014. RFID chips can operate in a completely passive power mode where the power needed to operate the RFID chip is drawn from the reader through inducing an electromagnetic current in the tag’s antenna. RFID chips have an internal energy harvesting unit that can produce up to 3 V to power up the RFID chip circuit. Moreover, the addition of sensing capabilities permits the integration of external sensors to the RFID chip and uses some of the harvesting energy as a supply to the sensor.

On the circuit level, the low power can be achieved by the energy efficient CMOS circuits.^271,272^ The total power in the CMOS technology is due to the dynamically dissipated power during the charging and discharging of the parasitic capacitances, the power dissipated during the time when both the NMOS and the PMOS conduct, and the losses due to a nonzero current of the MOS transistors in the off-state for digital circuits or to a biasing current for analog circuits. Investigating the energy efficiency of CMOS circuits will have the advantage of reducing the power consumption in data processing and control as well as for the data storage. Emerging technologies with low leakage power, including, for example, nonvolatile processors, ferroelectric random access memory (FRAM), and fully-depleted silicon-on-insulator (FD-SOI), and neural like computing are promising solutions for low power circuits.^273−275^ Besides, choosing the process technology with a high threshold voltage can further reduce leakage power. Another approach to reduce power consumption of the circuit is by using sleep mode in idle periods.^276^ In this approach, the reduction of the power consumption is achieved by software optimization. In this regard, different techniques such as dispersed electronics including edge processing, algorithm-architecture codesign, RF subsystems can be tailored for a wide range of future sensing applications.

## 5 Conclusions and Future Perspectives

The degradable materials and sensing technologies elaborated in this paper and their future evolution will play key roles in improving human health and realizing the goal of sustainable healthcare systems. Using biodegradable implantable and wearable sensors for continues monitoring of body condition could transform the future of disease management in healthcare systems toward preventative, predictive, and personalized management of diseases. In addition, the development of biodegradable electronic systems and sensors that naturally dissolve under ambient conditions could mitigate electronic waste problems.

The degradation rates of materials and dissolution behavior of bioresorbable devices in different biological systems can vary significantly. Engineered transient materials to control degradation at programmed rates are necessary for practical application. In addition, developing a high-performance and miniaturized power supply, circuits and chips on biodegradable substrates, and wireless transmission are required to develop a fully biodegradable platform. For future implantable sensors, the power consumption must be minimized, and devices might be able to take the required power from the movement or heat of the body or other *in situ* energy sources such as biofluid.

From the discussion in this paper, it is clear that the majority of reported degradable materials-based wearable devices focus on a single mode of analysis. However, multisensors with the combination of chemical and physical modes of sensing in a hybrid device or multifunctional devices that provide both monitoring and treatment would be needed in the future to obtain a more complete picture of the state of health of the user.

Finally, to realize these technologies for clinical uses, further understanding of biological systems and immune response to design the device specifically for *in vivo* environment is needed. To this end, greater interaction and collaboration between engineers and biologist are essential.
